# Disentangling the Relationship Between Urinary Metal Exposure and Osteoporosis Risk Across a Broad Population: A Comprehensive Supervised and Unsupervised Analysis

**DOI:** 10.3390/toxics12120866

**Published:** 2024-11-28

**Authors:** Jianing Liu, Kai Wang

**Affiliations:** 1 Medical Faculty, Ulm University, 89075 Ulm, Germany; 2 Medical School, Southeast University, Nanjing 210096, China

**Keywords:** environmental pollutants, bone health, machine learning, risk factor, mortality

## Abstract

**Background**: Limited evidence links urinary metal exposure to osteoporosis in broad populations, prompting this study to cover this knowledge gap using supervised and unsupervised approaches. **Methods**: This study included 15,923 participants from the National Health and Nutrition Examination Survey (NHANES) spanning from 1999 to 2020. Urinary concentrations of nine metals—barium (Ba), cadmium (Cd), cobalt (Co), cesium (Cs), molybdenum (Mo), lead (Pb), antimony (Sb), thallium (Tl), and tungsten (Tu)—were measured using inductively coupled plasma mass spectrometry (ICP-MS). Osteoporosis was assessed via dual-energy X-ray absorptiometry. A weighted quantile sum (WQS) regression analysis evaluated each metal’s contribution to osteoporosis risk. Partitioning around medoids (PAM) clustering identified the high- and low-exposure groups, and their association with the risk and prognosis of osteoporosis was evaluated. **Results**: WQS regression identified Cd as a significant osteoporosis risk factor in the general population (odds ratio (OR) = 1.19, 95% confidence interval (CI): 1.08, 1.31, weight = 0.66). Pb notably affected those individuals aged 30–49 years and classified as Mexican American, while Sb impacted Black individuals. PAM clustering showed that the high-exposure group had a significantly higher risk of osteoporosis (OR = 1.74, 95% CI: 1.43, 2.12) and cumulative mortality risk. **Conclusions**: Urinary metals are associated with the risk and prognosis of osteoporosis.

## 1. Introduction

Osteoporosis, a condition characterized by diminished bone mass and strength, the deterioration of bone tissue, and the disruption of bone microarchitecture [1,2], poses a significant public health challenge globally. This silent disease often progresses unnoticed until fractures occur, leading to severe morbidity, disability, reduced quality of life, and elevated mortality. Osteoporotic fractures, particularly of the hip and vertebrae, impose a substantial burden on healthcare systems due to the high cost of treatment and long-term care for fracture-related complications [3]. A recent meta-analysis indicated that the global prevalence of osteoporosis was 18.3%, highlighting the urgency of addressing this widespread condition [4]. While age, gender, hormonal status, and genetics are well-established risk factors for osteoporosis, increasing attention is being directed toward the role of environmental exposures in the risk of osteoporosis. Most existing research predominantly focuses on postmenopausal women and older adults, who are at a higher risk of osteoporosis due to hormonal changes and age-related bone loss [5,6,7,8]. However, younger populations are not immune to the impacts of osteoporosis [9]. Emerging evidence suggests that environmental factors can affect the risk of osteoporosis from a young age, potentially influencing peak bone mass and increasing susceptibility to osteoporosis later in life [10]. Studies have shown that even children and adolescents can experience bone density changes due to environmental exposures [11]. However, there is currently a lack of research exploring the impact of environmental pollution on osteoporosis across a broad population. Moreover, previous studies have often used values of bone mineral density (BMD) as the outcome measure [5,8,12], but BMD values can vary significantly depending on the population and measurement methods. Because of the lack of consistent diagnostic criteria and assessments of osteoporosis prevalence across different populations, the practical utility and generalizability of these studies are diminished. Under such circumstances, it becomes evident that more research is urgently needed—that not only focus on older adults but also encompasses a broader range of ages and populations. Such research would ideally employ international standards to diagnose osteoporosis based on reference values from homogeneous populations, providing more robust insights into the complex interplay between environmental exposures and the risk of osteoporosis.

Metals and metalloids [13,14,15,16], ubiquitous in the environment due to both natural occurrences and anthropogenic activities, have been implicated in various adverse health outcomes. Metals can accumulate in the human body over time, leading to chronic toxicity and contributing to conditions such as cardiovascular disease [17], cancer [18], miscarriages, and neurological disorders [19,20,21,22,23]. Although the impact of mixed metal exposure on osteoporosis is not fully understood, growing evidence suggests that metals and metalloids may have toxic effects on bone health [24]. For instance, cadmium (Cd) might interfere with calcium metabolism and bone remodeling processes [25]. Lead (Pb) could also disrupt calcium homeostasis, weakening cortical bone width [26]. High-dose exposure to cobalt (Co) has been implicated in affecting osteoblast proliferation, size, and shape, while concurrently elevating cellular oxidative stress levels [27]. Oxidative stress refers to a state of imbalance between the excessive production of reactive oxygen species (ROS) and the body’s antioxidant defense mechanisms. This imbalance is one of the critical contributors to the pathogenesis of osteoporosis, as it disrupts the delicate equilibrium required for bone remodeling. Specifically, oxidative stress is associated with increased osteoclast activity, inhibited osteoblast function, and impaired bone formation, all of which collectively lead to bone degradation and heightened fracture risk [28,29]. Although direct evidence linking certain metals to osteoporosis is lacking, many are known to induce oxidative stress. For instance, thallium (Tl) and antimony (Sb) have been documented to directly promote the generation of ROS [23,30], whereas barium (Ba), cesium (Cs), and tungsten (Tu) are associated with disruptions in ionic and metabolic homeostasis, potentially exacerbating oxidative stress at the cellular level [31,32]. These metals are prevalent in various industrial processes, consumer products, and environmental contaminants, making human exposure inevitable, so elucidating their relationship with bone health holds significant clinical relevance. However, the evidence is not entirely consistent. Some studies have found no significant association between metal exposure and the risk of osteoporosis outcomes [33], underscoring the imperative to elucidate these complex relationships.

Evaluating human exposure to metals is crucial for understanding their health impacts. Blood, urine, and hair are commonly used sampling methods for assessing metal exposure, with each offering specific benefits depending on the context. However, urinary metal evaluation holds distinct advantages in large-scale population screenings and preventive monitoring or follow-up. Firstly, urine collection is a noninvasive procedure that enhances participant compliance, particularly in populations unaware of their vulnerability to health risks, such as younger and middle-aged adults increasingly affected by lifestyle changes and pollution. The nature of urinary metal testing enables it to provide timely information about metal exposure and metabolism status, allowing individuals to retrospectively identify and mitigate potential exposure sources. Secondly, urine sampling does not require specialized medical personnel or equipment for collection, making it more cost-effective and feasible for use in large-scale epidemiological studies and routine screenings, particularly under limited resource settings. Thirdly, compared to hair samples, urine is also easier to handle and store, and there is less risk of sample contamination from environmental factors or cosmetic treatments, such as hair dye or shampoo. Furthermore, urinary metal concentrations have been established as significant biomarkers, exhibiting strong associations with various chronic conditions, including anemia [34], metabolic syndrome [35], diabetes [36], and cognitive disorder [37]. This evidence suggests their potential utility in predicting other chronic metabolic diseases such as osteoporosis.

Additionally, given the complexity of human exposure to multiple metals, it is essential to adopt an integrative approach that considers the individual and mixed effects of these metals on osteoporosis. In a large-scale analysis involving tens of thousands of individuals, the implementation of Bayesian kernel machine regression (BKMR) and Bayesian hierarchical models (BHMs) can be challenging due to their high-dimensional modeling structures, which require significant computational resources and result in longer convergence times, making them less feasible for large datasets [38]. Moreover, the output from these models can be difficult to interpret, especially when explaining mixed effects and their contributions to risk. On the other hand, methods like LASSO and ridge regression primarily focus on variable selection by applying regularization, but they fall short in providing a comprehensive assessment of the mixed effects of multiple exposures, as they tend to reduce the complexity of the model at the expense of capturing interactions and combined effects [39]. Therefore, we employed a dual analytical approach combining weighted quantile sum (WQS) regression and partitioning around medoids (PAM) clustering to assess the impact of metal mixtures on osteoporosis risk. The integrative approach offers a comprehensive and interpretable framework that captures both the overall mixture effect and the relative importance of each metal, simplifying communication with public health stakeholders and enhancing the clarity and actionability of the results. Details on the implementation of the WQS regression and PAM clustering are provided in Section 2.

In this study, we utilized a large, nationally representative dataset to explore the relationship between urinary metal exposure and osteoporosis through both supervised and unsupervised methodologies. This study’s objectives are threefold: First, we aim to examine the association between urinary metals and osteoporosis risk. Second, we seek to identify which specific metals within the mixed urinary exposures exert the greatest influence on this association and whether these effects vary across different subpopulations. Finally, we stratify the population by mixed urinary metal exposure levels within an unsupervised learning framework and validate this relationship by comparing osteoporosis risk and survival outcomes between high- and low-exposure groups, thereby providing a dual-layered confirmation of the robustness of these findings. By elucidating the associations between urinary metal exposure and osteoporosis risk, our findings may contribute to more targeted health education, inform screening strategies for high-risk groups in clinical practice, and support the refinement of public health policies and environmental regulations aimed at reducing exposure and mitigating related health risks.

## 2. Methods

### 2.1. Data Source and Study Population

This study utilized the National Health and Nutrition Examination Survey (NHANES) 1999 to 2020 pre-pandemic data, conducted by the National Center for Health Statistics (NCHS) of the Centers for Disease Control and Prevention (CDC). NHANES uses a stratified, multistage probability sampling design, ensuring that the study sample is nationally representative of the U.S. population across different regions, thereby minimizing concerns regarding regional exposure variability. The NHANES participants did not have documented histories of explicit metal contamination in their food sources [40,41]. Additionally, detailed information on the occupational distribution of participants is shown in Supplementary Figure S3.

The study population comprised individuals aged 8 years and older, with complete data on nine urinary metals—barium (Ba), cadmium (Cd), cobalt (Co), cesium (Cs), molybdenum (Mo), lead (Pb), antimony (Sb), thallium (Tl), and tungsten (Tu)—and sufficient information to diagnose osteoporosis. The selection of the nine metals included in this study was primarily based on the data availability and completeness in the NHANES dataset across multiple survey cycles from 1999 to 2020. These metals were consistently measured in a stratified subsample using standardized methods, ensuring data reliability and comparability over time. Additionally, their environmental ubiquity, potential public health relevance, possible links to the pathogenesis of osteoporosis, feasibility for detection using existing technologies, and potential as scalable biomarkers for future screening efforts were also important considerations in their inclusion.

The process of participant selection is shown in Supplementary Figure S1. Ultimately, a total of 15,923 participants were included in this study. In further survival analysis, participant vital status was determined by linking records to the National Death Index (NDI), with data on all-cause mortality collected until 31 December 2019. Although not all participants were included in the survival cohort analysis, Supplementary Table S1 shows that the baseline characteristics of the survival cohort are very similar to those of the total study population. This suggests that the survival analysis results are probably representative of the overall population’s prognosis.

### 2.2. Exposure Variables: Urinary Metals

The urinary concentrations of the nine metals were measured using inductively coupled plasma mass spectrometry (ICP-MS) at the CDC’s National Center for Environmental Health, employing PerkinElmer NexION 300D/350D or NexION 2000P models (PerkinElmer, Waltham, MA, USA) equipped with dynamic reaction cell (DRC) technology to minimize interferences. Detailed protocols, including performance checks, calibration procedures, quality assurance measures, detailed parameters, and test data, are documented in the NHANES official laboratory operation manual [42]. Supplementary Figure S5 provides scatter and box plots illustrating the original urinary metal concentration data (unweighted) for the study population (N = 15,923).

Urine samples were meticulously collected, processed, and stored at temperatures ranging from −30 °C to −20 °C until analysis. Samples were diluted 1:9 with 2% nitric acid to solubilize and stabilize the metals in solution. The analytical procedure included the use of internal standards (iridium and rhodium) to correct for matrix effects, signal drift, and other potential instrumental interferences. Certified reference materials traceable to the National Institute of Standards and Technology (NIST), including urine-specific standards, were utilized to ensure the matrix compatibility of calibration standards, thus ensuring high accuracy in the reported concentrations. Detection limits for each metal were rigorously validated and recalibrated daily, achieving precision within ±15% of nominal values under normal concentrations and ±20% at levels near the limit of detection (LOD). To further ensure accuracy and precision, daily quality control (QC) protocols included the use of blanks, low- and high-bench QC materials, and spiked urine samples, analyzed alongside study samples. Bench QC results were required to fall within ±3 standard deviations (SD) of the established means for the analytical run to be considered valid. Analytical performance was also verified through daily checks for analyte-to-internal standard ratio stability and instrument sensitivity, maintaining coefficients of variation (CVs) ≤ 15% across replicates. The instruments operated with argon as the carrier gas, ionizing metals in a plasma at temperatures between 4500 and 6500K. Analyses were conducted in both standard and DRC modes to address possible interferences and optimize detection for specific elements. Instrument stability was ensured with a warm-up period of 1–1.5 h prior to the analysis of samples, and analytical runs were monitored with rigorous internal checks, such as calibration curve accuracy (R^2^ ≥ 0.98) and low contamination from reagents.

In this analysis, the distributions of urinary metal concentrations were right-skewed, necessitating natural log transformations to normalize the data distribution and reduce skewness. To address the potential interference of urinary creatinine, two adjustment methods were common: dividing urinary metal concentrations by urinary creatinine and using urinary creatinine as a covariate in regression models. Prior analysis confirmed that both methods produce consistent results regarding the direction of effect and statistical significance [43]. To facilitate a more straightforward interpretation of the effects of both actual and log-transformed urinary metal concentrations, the latter adjustment method was utilized in this study. Additionally, creatinine levels in urine were measured using the Jaffé rate reaction method, where creatinine reacts with picrate in an alkaline solution to form a red creatinine–picrate complex. Detailed laboratory methodologies and quality control/quality assurance data are available on the NHANES website (https://www.cdc.gov/nchs/nhanes/index.htm, accessed on 10 August 2024).

### 2.3. Outcome Variables: Osteoporosis

Trained and certified radiologic technologists conducted dual-energy X-ray absorptiometry (DXA) scans using the Hologic QDR-4500A fan-beam densitometer (Hologic, Inc., Bedford, MA, USA) at the Mobile Examination Center (MEC) to obtain bone mineral density (BMD) values. All DXA data were analyzed using Hologic APEX version 4.0 software. Additional details regarding the DXA protocols and procedures are available on the NHANES website. In this study, BMD values from the femoral neck or lumbar spine were used to calculate T-scores and Z-scores for the diagnosis of osteoporosis. According to the World Health Organization (WHO) criteria, the formulas for these scores are as follows:

*T*-score = *(Measured BMD − Mean BMD of White women aged 20–29 years)*/

*Standard deviation of BMD for White women aged 20–29 years*

Z-score = *(Measured BMD − Mean BMD of the same age group, gender, and race group)*/

*Standard deviation of BMD for the same age group, gender, and race group*

For postmenopausal women and men aged 50 years and older, the T-score is used to diagnose osteoporosis. A T-score of ≤−2.5 indicates osteoporosis. Additionally, individuals with a T-score between −1.0 and −2.5 who have a history of fragility fractures can also be diagnosed with osteoporosis [44,45]. For all other groups (children, premenopausal women, and men aged 20–49 years), the Z-score is utilized. A Z-score of ≤−2.0 or a history of fragility fractures as mentioned in the interview questionnaire is considered indicative of osteoporosis [46].

### 2.4. Statistical Analysis

To ensure nationally representative results, we implemented a weighting methodology that aligns with the complex NHANES sampling design, following the NHANES Guidelines [47]. For this analysis, we applied the specific weights of the urinary metal subsample, which account for nonresponse, noncoverage, and unequal selection probabilities.

In this analysis, we integrated the supervised and unsupervised approaches to leverage the strengths of both methods, enabling a comprehensive assessment of mixed metal exposures and a robust understanding of their impact on osteoporosis risk. To evaluate the single effect of each urinary metal, we used weighted logistic regression analysis to examine the association between each urinary metal concentration (as a continuous variable) and the presence of osteoporosis, with odds ratios (ORs). Each metal concentration was also categorized into quartiles (Q1–Q4), with Q1 serving as the reference group. Restricted cubic spline (RCS) analysis was performed to explore potential nonlinear relationships between metal concentrations and osteoporosis risk. To control for potential confounding effects, the regression models were adjusted for several covariates, including urinary creatinine, age, sex, race/ethnicity, family poverty–income ratio (PIR), diabetes stage, general obesity, and central obesity status.

To assess the importance of each metal in the combined effects, we employed a weighted quantile sum (WQS) regression. WQS regression, a supervised method, estimates the combined effect of multiple metals on osteoporosis risk while assigning weights to each metal based on its contribution to the outcome and providing a ranking of the metals most strongly associated with the risk. This method allows us to understand the relative importance of each metal within a mixture, providing targeted insights for public health interventions [48]. The WQS method accounts for the correlated and covariant nature of metal exposure by creating an index based on the weighted sum of quantile-transformed metal concentrations. This analysis was conducted using 1000 bootstrap samples to ensure robust estimates. The WQS index was then included in the logistic regression model to evaluate its association with osteoporosis, adjusting for relevant covariates [49].

To classify populations based on combined exposure levels, PAM clustering was applied based on the concentrations of nine urinary metals. PAM clustering, an unsupervised method, classifies the study population into distinct exposure groups based on the concentrations of multiple urinary metals without prior knowledge of osteoporosis outcomes. This approach allows for an unbiased classification, better simulating the natural occurrence of disease influenced by exposure factors. Compared to other clustering algorithms, PAM is particularly suited for this study due to its robustness against noise and outliers [50], making it ideal for environmental exposure assessments. The silhouette width method was used to determine the optimal number of clusters [51]. Subsequently, the characteristics between the clusters were compared, and clusters with high and low urinary metal exposure levels were defined. After population segmentation based on different exposure clusters, it is advantageous for comparing population characteristics under varying exposure statuses, making it easier to subsequently follow up the prognosis based on the different exposure populations. In this analysis, weighted logistic regression was also utilized to evaluate the association between clusters of exposure and osteoporosis risk. Furthermore, all-cause mortality as related to metal exposure and osteoporosis was assessed, plotting cumulative risk curves of death.

Sensitivity analyses were conducted using the propensity score matching (PSM) method to reduce potential confounding and ensure the robustness of the results. This approach can balance the distribution of covariates between exposed and unexposed groups, thereby mimicking a randomized experimental design [52]. In this study, propensity scores were estimated using a logistic regression model that incorporated the following covariates: age, sex, race, family poverty–income ratio (PIR), urinary creatinine (mg/dL), diabetes status, general obesity, and central obesity. Matching was performed using a 1:1 nearest-neighbor approach without replacement and with a caliper width of 0.05 standard deviations of the logit of the propensity score.

### 2.5. Software and Statistical Significance

All statistical analyses were conducted using R software version 4.2.0 (R Core Team, R Foundation for Statistical Computing, Vienna, Austria). Statistical significance was set to a *p*-value of <0.05 (two-sided). Continuous variables were presented as the median (25th percentage, 75th percentage), while categorical variables were expressed as frequencies (N) and percentages (%). This study reported unweighted frequencies (N) to ensure transparency in reflecting the actual sample size involved in the study but demonstrated weighted percentages (%) to accurately represent the distribution of variables in the population, accounting for the complex survey design.

## 3. Results

### 3.1. Baseline Characteristics of the Study Population

Table 1 presents the baseline characteristics of the study participants (N = 15,923). The median age of the participants was 43 years, with a nearly equal distribution of males and females. The majority of the participants were White individuals. The median urinary creatinine level was 109 mg/dL. Approximately half of the participants were classified as overweight, and 45.7% were identified as presenting central obesity. Additionally, 9.97% of the participants had a history of diabetes. Osteoporosis was diagnosed in 12.67% of the participants. To facilitate a more intuitive understanding of the population characteristics and their proportional distribution, some of these data are also presented in a pie chart in Supplementary Figure S4.

### 3.2. Urinary Metal Profiles

**Figure 1:**
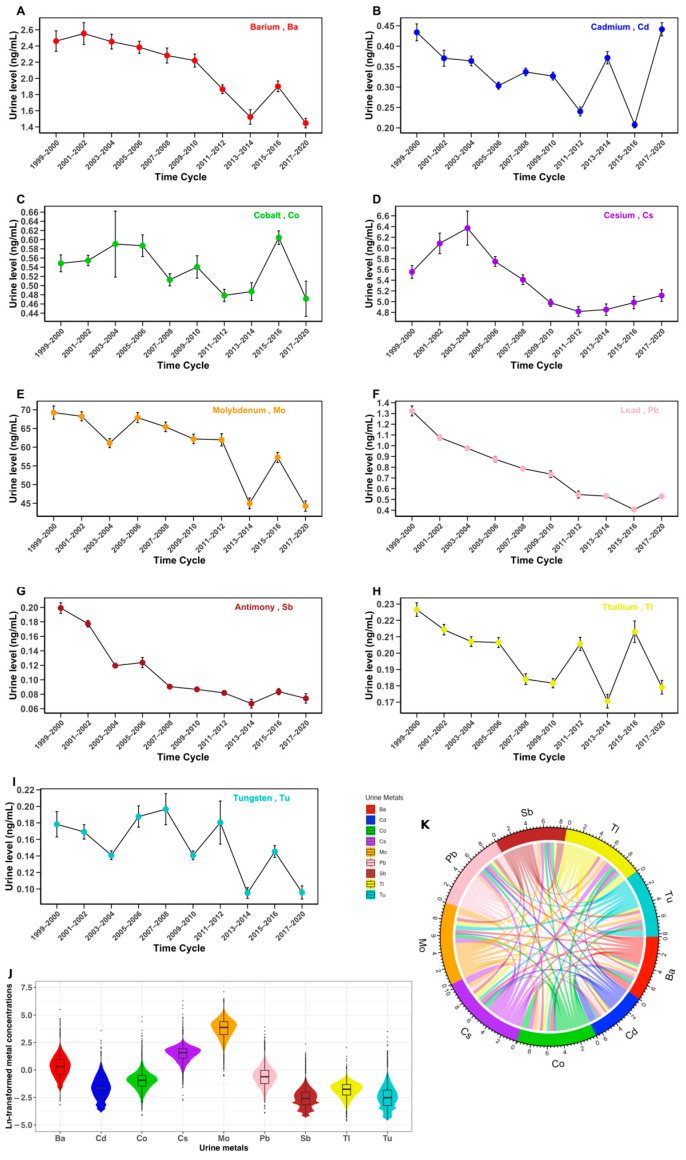
Overview of the data on the nine urinary metals. (**A**–**I**) Trends of the change in urinary metal concentrations from 1999 to 2020: (**A**) barium, Ba; (**B**) cadmium, Cd; (**C**) cobalt, Co; (**D**) cesium, Cs; (**E**) molybdenum, Mo; (**F**) lead, Pb; (**G**) antimony, Sb; (**H**) thallium, Tl; (**I**) tungsten, Tu. (**J**) Ln-transformed urinary metal concentration distribution among study participants. (**K**) Correlations between the ln-transformed urinary concentrations of the nine metals among the study population.

The temporal trends of the nine urinary metals from 1999 to 2020 are illustrated in Figure 1. Over this nearly two-decade span, the concentrations of Ba, Cs, Pb, and Sb generally showed a decreasing trend. In contrast, the concentrations of the remaining metals exhibited varying degrees of fluctuation over time. Significance tests comparing urinary metal concentrations across time points are presented in Supplementary Tables S5–S13, providing statistical evidence for the observed temporal differences illustrated in Figure 1. Additionally, the violin chart provides a visual representation of their distribution in urine, highlighting that Mo has a substantially higher urinary concentration compared to the other eight metals. Moreover, the chord chart reveals correlations among the nine urinary metal compounds. The Spearman correlation coefficients for urinary metals ranged from 0.12 to 0.79, with the strongest correlation observed between cesium (Cs) and thallium (Tl).

### 3.3. Association of Each Urinary Metal with Osteoporosis Risk

**Figure 2:**
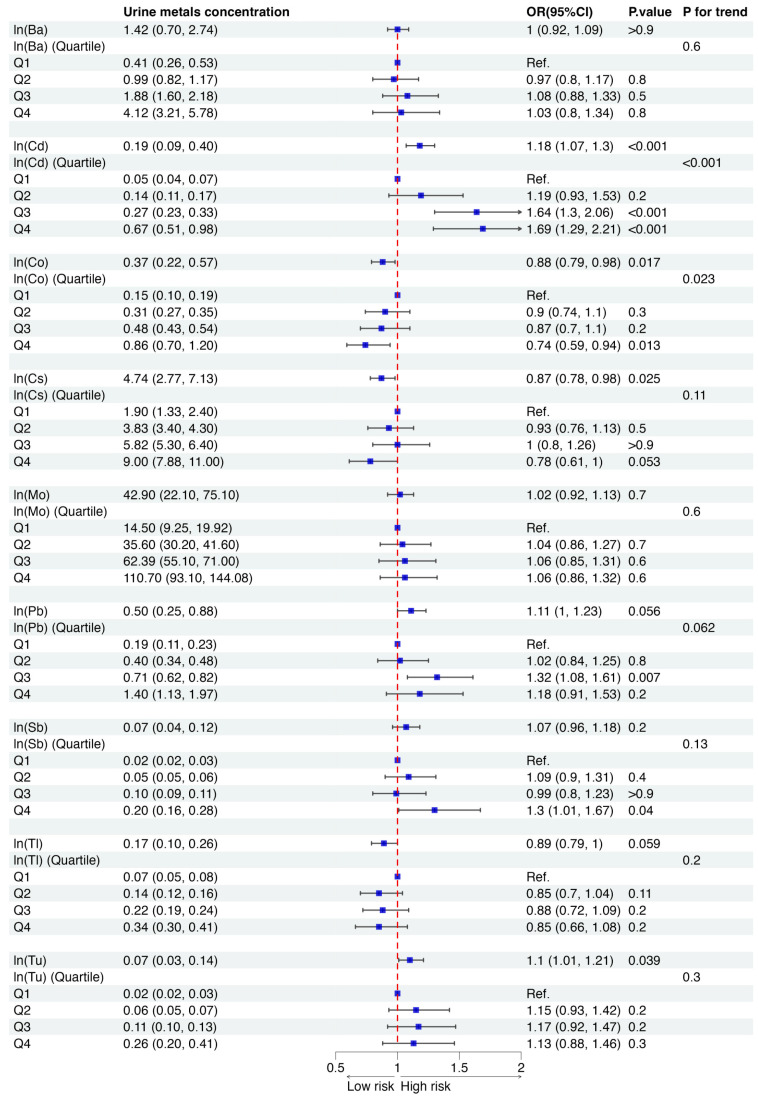
Associations between each urine metal concentration and osteoporosis risk. Note: Models with adjustment for urine creatinine, age, sex, race/ethnicity, poverty–income ratio (PIR), diabetes, general obesity, and central obesity.

Figure 2 illustrates the associations between individual urinary metal concentrations and osteoporosis using multivariable weighted logistic regression. Significant positive associations were observed for urinary Cd and Tu with the likelihood of osteoporosis. Additionally, participants in the highest quartile (Q4) of urinary Sb concentration and the third quartile (Q3) of urinary Pb concentration exhibited significantly higher risks of osteoporosis compared to those in the lowest quartile (Q1). Intriguingly, urinary Co and Cs demonstrated negative associations with osteoporosis risk in this study population. Furthermore, the RCS analysis (Supplementary Figure S2) indicated no significant nonlinear dose-response relationships between urinary metal concentrations and osteoporosis outcomes (all P_nonlinear_ > 0.05).

**Figure 3:**
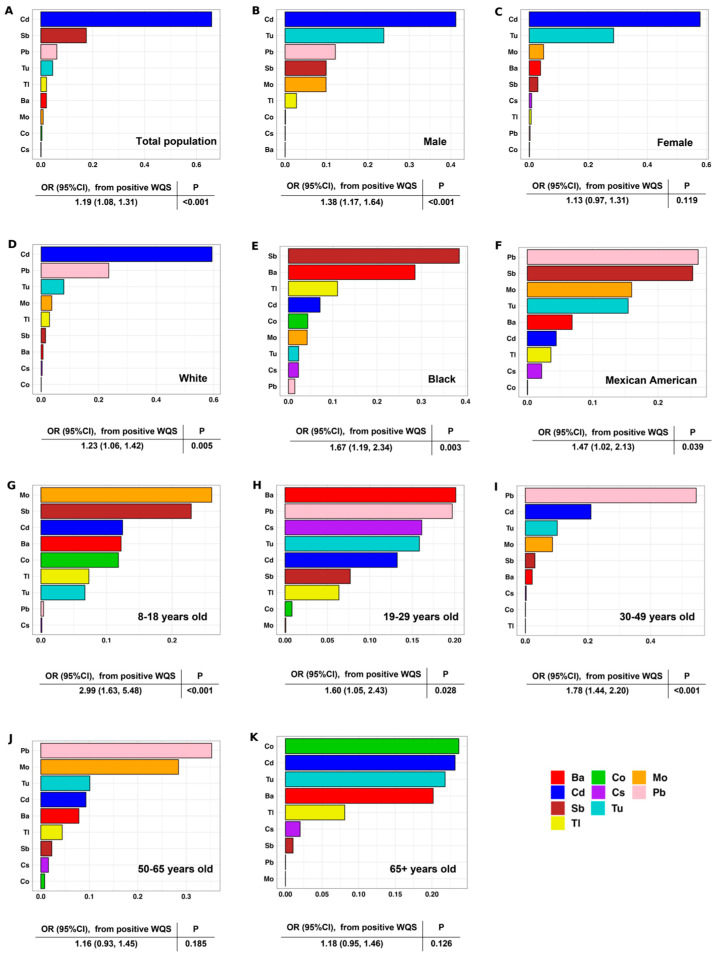
Weights of urinary metal mixtures based on WQS regression associated with osteoporosis in diverse populations. (**A**) Total population; (**B**) Male; (**C**) Female; (**D**)White; (**E**) Black; (**F**) Mexican American; (**G**) 8–18 years old; (**H**) 19–29 years old; (**I**) 30–49 years old; (**J**) 50–65 years old; (**K**) 65+ years old. Note: Models with adjustment for urine creatinine, age, sex, race/ethnicity, poverty–income ratio (PIR), diabetes, general obesity, and central obesity, except the stratification factor for the related subpopulations.

Notably, Figure 3 showcases the WQS regression results, highlighting the relative weights of urinary metals in contributing to osteoporosis risk across the total population and various demographic subgroups. For the total population, we observed that with each doubling of the WQS index, the odds of developing osteoporosis increased by 1.19 times. Among the nine metals, urinary Cd had the greatest impact on increasing osteoporosis risk (weight of Cd = 0.66), followed by Sb and Pb. We also assessed subpopulations with diverse demographic characteristics. Urinary Cd was the top contributor regardless of gender, while urinary Pb was an important contributor in the young and middle-aged groups (weight of Pb = 0.20, for 19–29 years; weight of Pb = 0.55, for 30–49 years). For Black individuals, urinary Sb had the greatest influence on osteoporosis risk, with a weight of 0.39. Among Mexican Americans, urinary Pb emerged as the most impactful metal, carrying a weight of 0.26. Conversely, urinary Cd was the most significant contributor to osteoporosis risk in White individuals, with a weight of 0.59.

### 3.4. Identification of Different Mixed Metal Exposure Groups Using PAM Clustering

**Figure 4:**
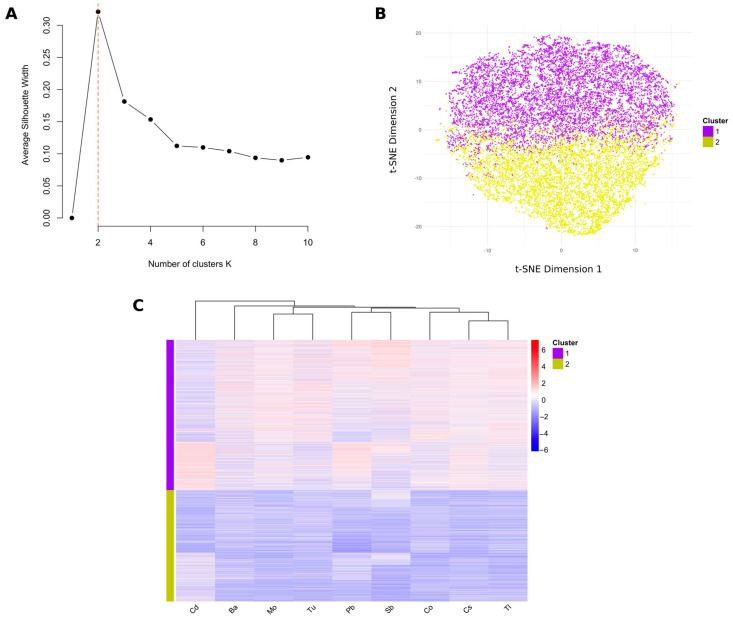
Overview of results from the PAM clustering for the 15,923 study participants based on urinary metals. (**A**) Assessment of the best number of clusters by the silhouette method. (**B**) t-SNE visualization of PAM clustering. (**C**) Heat maps for the ln-transformed concentrations of the nine urinary metals grouped by clusters.

To identify clusters with different exposure statuses among the 15,923 participants in this study, we employed PAM clustering based on the nine urinary metal concentrations. The silhouette method was used to determine the optimal number of clusters, with the average silhouette width peaking at two clusters, indicating that this was the most appropriate number of clusters for this analysis (Figure 4A). The t-distributed stochastic neighbor embedding (t-SNE) plot provides a visualization of the PAM clustering results (Figure 4B). The heat map in Figure 4C illustrates the ln-transformed concentrations of the nine urinary metals, grouped by the two clusters identified. Cluster 1 (purple) shows higher concentrations of urinary metals, while Cluster 2 (yellow) exhibits lower levels. Consequently, Cluster 1 was referred to as the high-exposure group and Cluster 2 as the low-exposure group. Supplementary Table S2 shows that 6786 individuals were classified into the low-exposure group, while 9137 individuals were classified into the high-exposure group, with significant differences in the basic characteristics between the groups. Further evaluation of metal concentrations, as shown in Table 2, indicates that the level of urinary metals in the high-exposure group was 2.20 to 3.16 times higher than that in the low-exposure group.

### 3.5. Association Between the Exposure Level of Urinary Mixed Metals and Osteoporosis Risk

**Figure 5:**
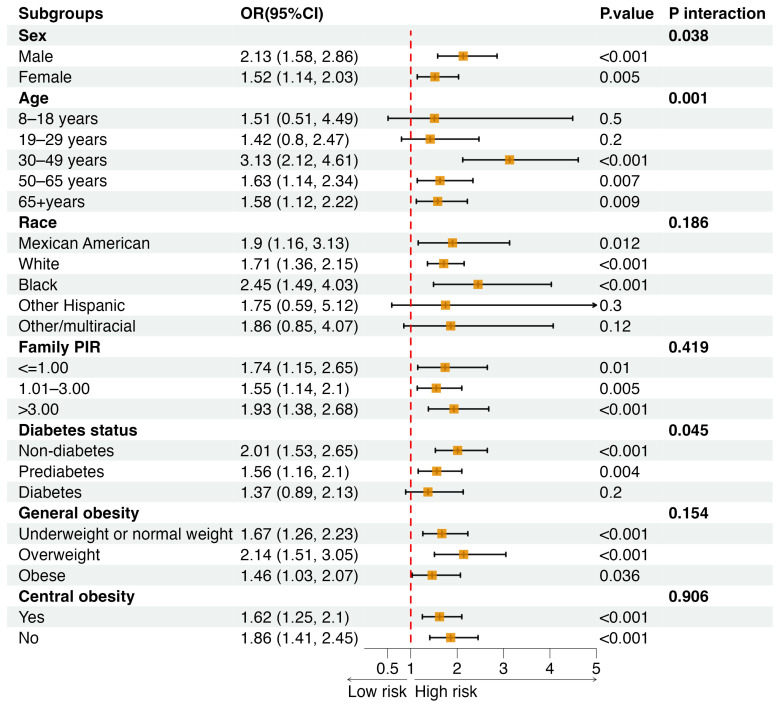
Subgroup analysis for the associations of urinary metal exposure clusters (high vs. low) with osteoporosis. Note: Models with adjustment for urine creatinine, age, sex, race/ethnicity, poverty–income ratio (PIR), diabetes, general obesity, and central obesity.

We employed three adjustment models to conduct a weighted logistic regression analysis examining the relationship between the exposure clusters identified by PAM clustering and osteoporosis, with the low-exposure group serving as the reference level. The fully adjusted model in Table 3 indicated that, compared to the low-exposure group, the high-exposure group had a 1.74 times higher likelihood of developing osteoporosis (95% CI: 1.43, 2.12). The subgroup analysis (Figure 5) revealed that the association between high metal exposure and osteoporosis risk was consistent across most subgroups, with particularly notable risks observed in males, middle-aged adults (30–49 years), Black (African ancestry) participants, or non-diabetic participants.

To further validate our findings, we performed a sensitivity analysis using propensity score matching (PSM), simulating the design and conditions of a prospective cohort study. When all basic characteristics were comparable between the high- and low-exposure groups after PSM, the osteoporosis prevalence was 17.46% in the high-exposure group and 9.59% in the low-exposure group, indicating a significant difference (Supplementary Table S3). We also used osteopenia, a precursor to osteoporosis, as the outcome variable, and the results remained consistent (Supplementary Table S4).

**Figure 6:**
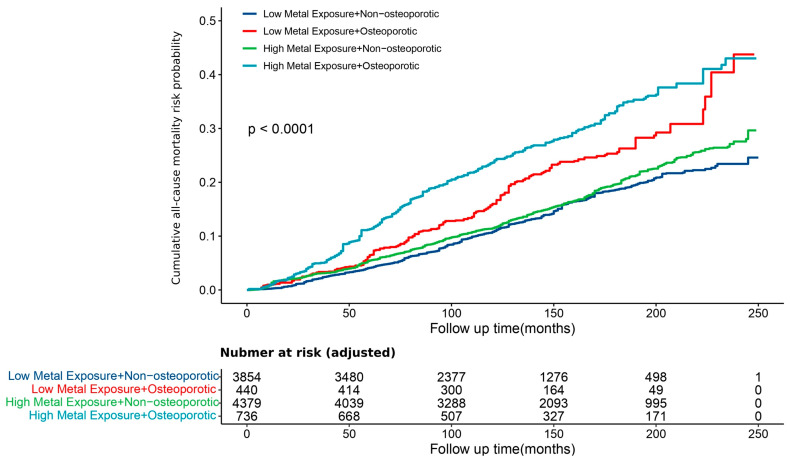
Cumulative all-cause mortality risks by urinary metal exposure level and osteoporosis status. Note: (1) The curves were adjusted for urine creatinine, age, sex, race/ethnicity, poverty–income ratio (PIR), diabetes, general obesity, and central obesity using inverse probability weighting. (2) The participant number in the risk table was from adjustment via inverse probability weighting, with extremes trimmed at the 99th and 1st percentiles to reduce the influence of outliers. (3) *p* values were derived from the log-rank test.

In terms of long-term prognosis, we analyzed survival cohorts to assess the cumulative risk of all-cause mortality. As demonstrated by the cumulative mortality risk curves, the difference in mortality risk between the high and low urinary metal exposure levels was negligible among the non-osteoporotic population. However, among the osteoporotic population, the high-exposure group exhibited a significantly higher mortality cumulative risk compared to the low-exposure group (Figure 6). This finding highlights that metal exposure significantly affects the survival outcomes of individuals with osteoporosis.

## 4. Discussion

This study employed a dual analytical approach, integrating both supervised and unsupervised methods, and revealed the association between urinary metal exposure and osteoporosis. Specifically, supervised analysis using weighted logistic regression and WQS regression identified urinary Cd as having the strongest impact on osteoporosis risk in the general population, while urinary Pb was particularly influential for individuals under 50 years of age and those classified as Mexican American. Urinary Sb exhibited the most significant effect among Black individuals, whereas urinary Cd was the key contributor among White individuals. These findings were further validated by unsupervised PAM clustering, which demonstrated that individuals with high urinary metal exposure had a significantly higher risk of osteoporosis compared to those with lower exposure. Additionally, metal exposure, particularly in osteoporotic patients, was associated with an elevated risk of all-cause mortality. These results underscore the necessity for demographic-specific prevention strategies and highlight the critical importance of metal control to improve bone health and long-term health outcomes.

Our findings, based on a nationally representative, multi-ethnic U.S. cohort, align with results from other population-based studies examining the role of Cd in bone health. For instance, Van Larebeke et al. in a Belgian cohort identified a significant association between increased urinary Cd levels and osteoporosis, highlighting Cd’s detrimental impact on bone health [53]. Similarly, Lim et al. found that increased blood Cd and Pb levels correlated with decreased bone mineral density in a South Korean cohort [12]. Additionally, Lv et al. reported higher blood Cd levels in a Chinese population, significantly increasing osteoporosis risk [54]. Mechanistically, the detrimental effects of Cd on bone tissue can be explained through insights from molecular biology, biochemistry, and toxicology. Firstly, Cd exerts detrimental effects on bone health through its complex interactions with calcium (Ca) metabolism and cellular functions. Metals might interact with Ca through their differential affinities for cellular ligands, based on the “hard and soft acid-base” (HSAB) framework [55,56]. Hard metal ions, such as calcium (Ca) and magnesium (Mg), preferentially bind to oxygen-containing ligands, directly supporting bone mineralization and structural integrity. In contrast, Cd, as a soft metal ion with a high affinity for sulfur-containing ligands like thiols, can compete with calcium for binding sites due to the non-specific nature of many cellular and systemic binding sites. This competitive displacement disrupts calcium’s critical role in bone metabolism and structural stability, particularly under conditions of oxidative stress or metabolic imbalance [57]. Cd can disrupt calcium absorption in the gastrointestinal tract and renal calcium reabsorption, reducing systemic calcium availability. These disruptions compromise calcium homeostasis, impair bone mineralization, and destabilize bone architecture, significantly increasing the risk of reduced bone density, fractures, and osteoporosis [58]. Secondly, Cd induces oxidative stress by depleting glutathione (GSH) and increasing reactive oxygen species (ROS), which damage osteoblasts (bone-forming cells) and promote osteoclast-mediated bone resorption [59,60,61]. This oxidative imbalance inhibits bone formation and accelerates bone loss. Concurrently, Cd directly downregulated osteogenic differentiation genes via the RANKL/OPG signaling pathway, resulting in abnormal bone formation and bone resorption [62]. Additionally, Cd affects the parathyroid hormone (PTH)–vitamin D axis, reducing vitamin D activation in the kidneys [25]. This leads to impaired calcium homeostasis and reduced bone mineralization. It is noteworthy that among the multiple metals studied, Cd stands out as the most impactful on osteoporosis. This pronounced effect might be attributed not only to its unique mechanisms but also to its prolonged biological half-life, high bioavailability, and potent biochemical interactions [63,64]. Collectively, these factors enable Cd to disrupt bone remodeling more effectively than other metals, ultimately leading to osteoporosis. Hence, the significance of our study lies in adding further evidence by demonstrating a strong association between urinary Cd levels and osteoporosis in a large U.S. population, reinforcing Cd as a biomarker for osteoporosis risk. However, establishing a definitive causal relationship will require further longitudinal analyses, intervention studies, and mechanistic investigations.

From the perspective of the affected population, our research reveals that the association between urinary metal concentrations and osteoporosis is particularly significant in the 30–49 age group, alongside the traditionally recognized high-risk group of individuals aged 50 and above. This finding is crucial, given the increasing prevalence of osteoporosis among younger populations [65]. Bone density typically reaches its peak in early adulthood and gradually begins to decline after middle age [66], highlighting the importance of early intervention in younger demographics to prevent the onset of osteoporosis. From an individualized prevention perspective, our findings indicate that Pb in urine poses a significant risk for osteoporosis among those aged 30–49. This association between Pb and osteoporosis aligns with results from prior studies [67,68]. Pb exposure is a common occurrence in daily life, stemming from various sources such as paint, contaminated dust and soil, drinking water, lead-glazed ceramics, toys, cosmetics, and herbal remedies [69]. While Pb shares some mechanistic similarities with Cd, such as inducing oxidative stress and interfering with vitamin D metabolism [70,71], its impact on osteoporosis is more specifically concentrated in bone tissue. Due to its similar charge density to calcium, Pb can replace calcium in hydroxyapatite, the primary mineral component of bone. This substitution compromises bone structure, leading to reduced density and diminished strength [72]. In vitro studies also suggest that lead can inhibit the function of osteoblasts and growth plate chondrocytes, disrupting endochondral bone formation and leading to skeletal abnormalities [73]. Preventing Pb exposure could therefore play a vital role in reducing osteoporosis incidence and particularly improving bone health outcomes in younger populations.

Unexpectedly, our study indicates a potential protective effect of Co on osteoporosis, but it can be supported by previous experimental evidence. Studies have shown that Co can enhance osteogenesis and angiogenesis, promoting bone formation through the upregulation of hypoxia-inducible factor 1-alpha (HIF-1α), BMP-2, and Runx2 expression. This is particularly beneficial at low doses, which stimulate collagen type 1 production and support bone regeneration [74]. However, high doses of Co can have adverse effects, including promoting osteoclast genesis and bone resorption by affecting the RANKL/OPG ratio, as well as inducing oxidative stress and inflammation [75]. Overall, this protective effect observed in our study suggests that Co’s role in bone metabolism warrants further investigation to better understand its dose-dependent impacts and potential therapeutic applications in preventing and treating osteoporosis.

Notably, our study explored the prognostic implications of metal exposure on the long-term outcomes in osteoporotic patients. We found that the survival outcome of osteoporotic individuals was significantly influenced by their urinary metal exposure levels, with a higher exposure correlating to an increased cumulative all-cause mortality risk. This observation suggests a complex interaction between osteoporosis and metal exposure, affecting long-term survival. Previous research has shown that metals such as Cd can exacerbate chronic conditions and contribute to higher mortality rates [76]. It has also been reported that osteoporotic fractures are linked to an elevated risk of all-cause mortality. This increased mortality risk is not only immediate, following a fracture, but can persist for several years thereafter [77,78]. By incorporating these findings, our research underscores the need for comprehensive public health strategies to reduce metal exposure, particularly in vulnerable populations like those with osteoporosis, as this may improve long-term health outcomes and lower mortality rates in this high-risk group.

Several limitations should be acknowledged. Firstly, the cross-sectional design precludes establishing causality between urinary metal exposure and osteoporosis. Secondly, our study, constrained by objective limitations, included only nine metals. Future research should incorporate a broader range of metals to deepen the understanding of the relationship between urinary metals and bone health. Thirdly, potential confounders such as dietary factors, lifestyle habits, and occupational exposures were not fully accounted for, which could influence the observed associations. The reliance on self-reported data for certain variables can also introduce the possibility of reporting bias, potentially affecting the accuracy of our findings. Fourthly, an important limitation is the absence of geographic and ecological data, such as participants’ proximity to contamination sources, availability of energy resources, or local environmental policies, which may influence exposure levels. As NHANES data lack location-specific context, assessing the impact of these factors is challenging. Future studies should integrate geographic and ecological variables to better understand how environmental factors affect metal exposure and related health risks. Additionally, while the WQS regression and PAM clustering methods in this study did not increase the risk of multiple comparisons, Type I errors cannot be fully eliminated due to due to the complex interactions of the metal mixtures studied. As such, caution is required when interpreting the results. Moreover, the absence of a real-time pH measurement in urine samples represents another limitation, as pH can affect the bioavailability and chemical state of metals. Given that pH is time-sensitive and subject to changes during the freezing and thawing processes—an unavoidable aspect of large-sample investigations—it may no longer accurately reflect the original sample conditions. Future research should prioritize measuring urine pH immediately upon collection to enhance the reliability of findings related to metal exposure and osteoporosis risk. Lastly, although urinary metal concentrations were prioritized in this study due to their noninvasive nature and practicality in large-scale epidemiological research, there are certain limitations to their use as biomarkers for metal exposure. The accuracy of urinary measurements varies depending on the type of metal. Even after adjusting for urinary creatinine levels, urinary metal concentrations can still be influenced by factors such as kidney function, circadian rhythm variations, and high inter-individual variability at low-exposure levels [79,80]. Future studies should consider incorporating multiple biological matrices, such as blood, hair, or bone, to provide a more comprehensive understanding of both short- and long-term metal exposures and their relationship to osteoporosis risk. Validating urinary metal levels against these biological matrices would help establish their reliability as biomarkers of exposure. Additionally, mechanistic research exploring the cellular and molecular pathways linking specific metals to bone health is essential for understanding causality. Longitudinal and interventional studies assessing the impact of reducing metal exposure on osteoporosis prevention are also critical to advancing this field.

Our study suggests several implications for patients, healthcare providers, and policy makers in addressing the relationship between metal exposure and osteoporosis. For patients, especially those at risk of osteoporosis, the routine screening of urinary metal concentrations can be a valuable tool for early detection. This noninvasive approach can detect elevated exposure levels, which could otherwise go unnoticed, thereby allowing for earlier intervention before the clinical onset of osteoporosis. For healthcare providers, it is crucial to incorporate metal exposure into risk assessments for osteoporosis, especially in demographics traditionally viewed as lower risk, such as middle-aged adults. Additionally, considering the unique effects of metal exposure across different ages, genders, and ethnic backgrounds, personalized screening, monitoring, and follow-up strategies should be adopted to ensure that patients receive appropriate interventions based on their risk profiles. For policymakers, our findings emphasize the importance of implementing stricter environmental regulations to limit metal exposure. Given the strong associations between metal exposure, osteoporosis, and increased mortality risk, reducing environmental contamination—particularly through targeted policies on industrial emissions and public awareness campaigns—could reduce the disease burden and improve public health outcomes.

## 5. Conclusions

Our study demonstrates a significant association between urinary mixed metal exposure and the risk of osteoporosis, as well as long-term survival outcomes. By integrating both supervised and unsupervised analytical methods, we comprehensively assessed both the individual and combined effects of multiple metals. Among the mixed metal exposures, cadmium (Cd) emerged as the most influential factor, while lead (Pb) and antimony (Sb) exhibited pronounced effects within specific ethnic subgroups. Utilizing a large, nationally representative sample and including a broad age range—from children to older adults—our findings highlight the relevance of metal exposure to osteoporosis risk across the lifespan, extending beyond older adults to younger and middle-aged populations. These results underscore the critical importance of implementing metal mitigation strategies and incorporating targeted health screenings into osteoporosis prevention efforts. Addressing environmental metal exposure may allow for the more effective management of osteoporosis risk and associated health outcomes across diverse populations.

## Figures and Tables

**Table 1:** Basic characteristics of the study participants (N = 15,923).

Characteristic ^2^	Median (25th, 75th) or N (%) ^1^
Age, years	43.0 (25.0, 56.0)
Sex, N (%)	
Female	7554 (49.38%)
Male	8369 (50.62%)
Race, N (%)	
White	6567 (69.17%)
Black	3739 (11.12%)
Mexican American	3190 (7.97%)
Other Hispanic	1202 (5.58%)
Other/multiracial	1225 (6.16%)
Family poverty–income ratio (PIR)	2.93 (1.48, 4.95)
Marital status, N (%)	
Married/Living with Partner	6953 (54.46%)
Widowed/Divorced/Separated	2446 (15.56%)
Never married	6524 (29.98%)
Urinary Creatinine, mg/dL	109.00 (61.00, 169.00)
Diabetes status, N (%)	
Non-diabetes	9849 (62.37%)
Prediabetes	4232 (27.66%)
Diabetes	1842 (9.97%)
General obesity (based on BMI, kg/m^2^), N (%)	
Underweight (<18.5)	1483 (6.29%)
Normal (18.5 to <25)	5463 (32.83%)
Overweight (25 to <30)	4705 (31.95%)
Obese (30 or greater)	4272 (28.93%)
Central obesity, N (%)	6595 (45.70%)
Barium (Ba), ng/mL	1.42 (0.70, 2.74)
Cadmium (Cd), ng/mL	0.19 (0.09, 0.40)
Cobalt (Co), ng/mL	0.37 (0.22, 0.57)
Cesium (Cs), ng/mL	4.74 (2.77, 7.13)
Molybdenum (Mo), ng/mL	42.90 (22.10, 75.10)
Lead (Pb), ng/mL	0.50 (0.25, 0.88)
Antimony (Sb), ng/mL	0.07 (0.04, 0.12)
Thallium (Tl), ng/mL	0.17 (0.10, 0.26)
Tungsten (Tu), ng/mL	0.07 (0.03, 0.14)
Osteoporosis, N (%)	1683 (12.67%)

^1^ N is unweighted, while percentages (%) and medians (25th, 75th) are weighted. ^2^ Median (25th, 75th) for continuous; N (%) for categorical. Abbreviations: BMI, body mass index.

**Table 2:** Comparison of the concentrations of urinary metals between the high-exposure and low-exposure clusters.

Urinary Metals	Concentration Ratio	t	*p*-Value
Ba	2.44	30.77	<0.001
Cd	2.20	28.18	<0.001
Co	2.54	22.15	<0.001
Cs	2.34	42.86	<0.001
Mo	2.90	81.29	<0.001
Pb	2.84	48.02	<0.001
Sb	2.71	35.88	<0.001
Tl	2.31	76.02	<0.001
Tu	3.16	21.46	<0.001

Note: Concentration Ratio = concentration in the high-exposure group/concentration in the low-exposure group (Cluster 2).

**Table 3:** Associations of urinary metal exposure clusters (high vs. low) identified by PAM clustering with osteoporosis.

	N *	OR	95%CI	*p* Value
Model 1 ^1^	15,923	2.01	(1.67, 2.41)	<0.001
Model 2 ^2^	15,923	1.80	(1.48, 2.19)	<0.001
Model 3 ^3^	15,923	1.74	(1.43, 2.12)	<0.001
Sensitivity analysis—PSM ^4^	4850	1.99	(1.59, 2.50)	<0.001

Note: * N is unweighted. ^1^ Model 1: Adjusted for urine creatinine. ^2^ Model 2: Adjusted for urine creatinine, age, sex, race/ethnicity, and poverty–income ratio (PIR). ^3^ Model 3: Additional adjustments included diabetes, general obesity, and central obesity. ^4^ PSM was utilized to ensure no statistical differences between the low-exposure and high-exposure clusters in the following variables: urine creatinine, age, sex, race/ethnicity, poverty–income ratio (PIR), diabetes, general obesity, and central obesity. A univariate logistic regression model was conducted afterwards to calculate the OR. Abbreviations: OR, odds ratio; 95% CI, 95% confidence interval.

## Data Availability

The datasets used and/or analyzed during this study are available from the National Health and Nutrition Examination Survey (NHANES) database (https://www.cdc.gov/nchs/nhanes/index.htm, accessed on 10 August 2024).

## Supplementary Materials

The following supporting information can be downloaded at: https://www.mdpi.com/article/10.3390/toxics12120866/s1, Figure S1: Flow chart of the participant selection; Figure S2: Restricted cubic spline plots for the non-linear dose-response relationship of each urinary metal concentration with osteoporosis risk; Figure S3: The industry distribution of participants in this study; Figure S4: Pie charts of the study participants’ characteristics; Figure S5: Scatter and box plots illustrating the original urine metal concentration data (unweighted data) (*N* = 15,923); Table S1: Baseline characteristics of total population and population included in the survival cohort; Table S2: Basic characteristics of participants from two clusters (low-exposure and high-exposure group) identified by PAM clustering (*N* = 15,923); Table S3: Comparison in characteristics of participants from two clusters (low-exposure and high-exposure group) identified by PAM clustering after PSM (N = 4850); Table S4: Associations of urinary metal exposure clusters (high vs low) identified by PAM clustering with osteopenia; Table S5: Results of significance tests for temporal differences in urinary barium (Ba) concentrations (1999–2020); Table S6: Results of significance tests for temporal differences in urinary cadmium (Cd) concentrations (1999–2020); Table S7: Results of significance tests for temporal differences in urinary cobalt (Co) concentrations (1999–2020); Table S8: Results of significance tests for temporal differences in urinary cesium (Cs) concentrations (1999–2020); Table S9: Results of significance tests for temporal differences in urinary molybdenum (Mo) concentrations (1999–2020); Table S10: Results of significance tests for temporal differences in urinary lead (Pb) concentrations (1999–2020); Table S11: Results of significance tests for temporal differences in urinary antimony (Sb) concentrations (1999–2020); Table S12: Results of significance tests for temporal differences in urinary thallium (Tl) concentrations (1999–2020); Table S13: Results of significance tests for temporal differences in urinary tungsten (Tu) concentrations (1999–2020).
