# Supplementary material for: Disentangling the Relationship Between Urinary Metal Exposure and Osteoporosis Risk Across a Broad Population: A Comprehensive Supervised and Unsupervised Analysis
Source: Toxics. 2024 Nov 28;12(12):866. doi: 10.3390/toxics12120866 (PMC11679131; doi:10.3390/toxics12120866)
Supplement: Supplementary file 1 [file toxics-12-00866-s001.zip › toxics-3200191-supplementary.pdf]

Supplementary Figures and Tables

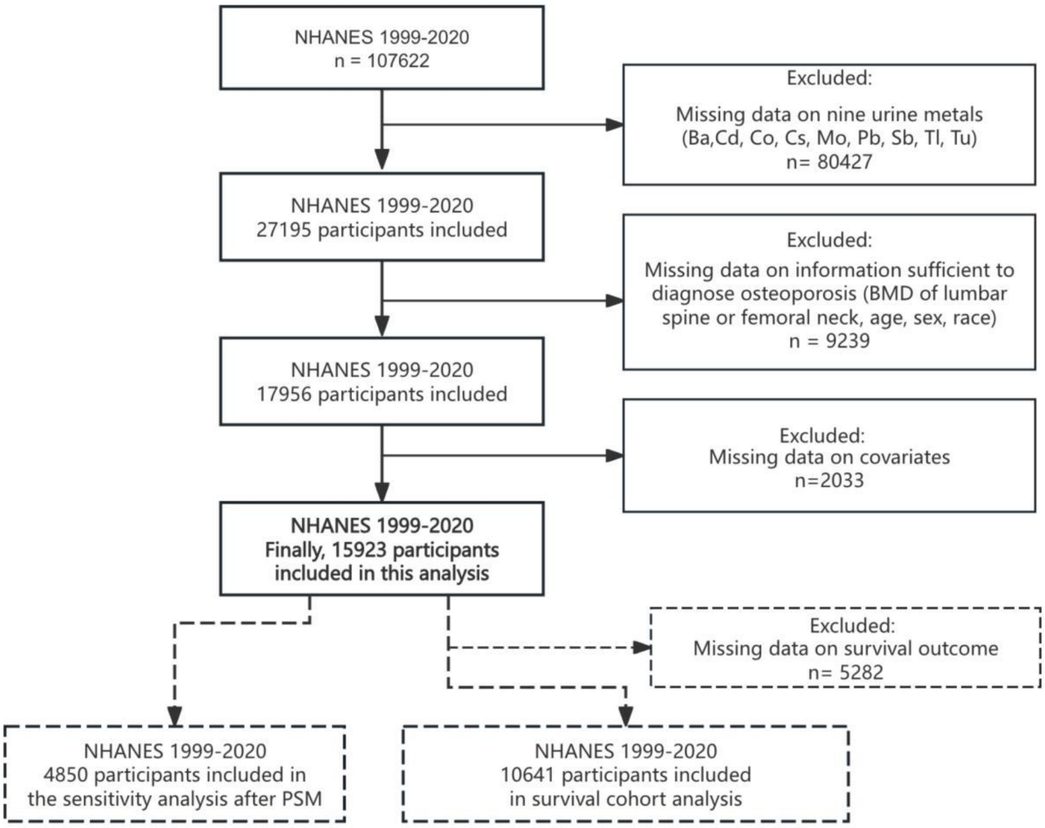

Supplementary Figure S1. Flow chart of the participant selection.

Abbreviations: Barium, Ba; Cadmium, Cd; Cobalt, Co; Cesium, Cs; Molybdenum, Mo; Lead, Pb; Antimony, Sb; Thallium, Tl; Tungsten, Tu; Bone Mineral Density， BMD; Propensity Score Matching, PSM.

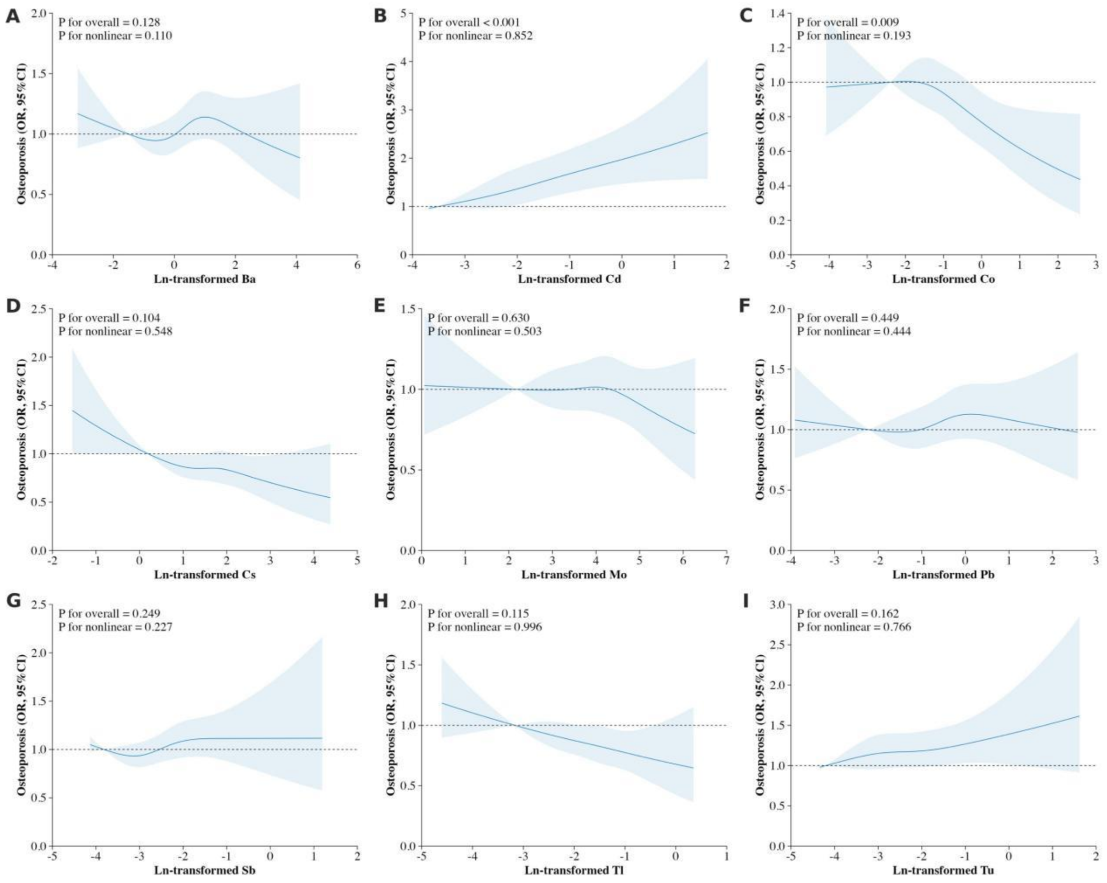

Supplementary Figure S2. Restricted cubic spline plots for the non-linear dose-response relationship of each urinary metal concentration with osteoporosis risk. (A) Barium, Ba; **(B)** Cadmium, Cd; **(C)** Cobalt, Co; **(D)** Cesium, Cs; **(E)** Molybdenum, Mo; **(F)** Lead, Pb; **(G)** Antimony, Sb; **(H)** Thallium, Tl; **(I)** Tungsten, Tu. *Note: Models with adjustment for urine creatinine, age, sex, race/ethnicity, and poverty income ratio (PIR), diabetes, general obesity, and central obesity. Multivariable-adjusted OR (blue solid lines) and 95% CI (shadow) for the association between urinary metal concentration and osteoporosis risk. The k1 point was designated as reference point (y=0)*

Supplementary Table S1. Baseline characteristics of total population and population included in the survival cohort.

Characteristic [2]	Total population N = 15923 (100%)[1]	Population included in the survival cohort N = 10641 (77%)[1]
Metals Exposure Clusters		
Low metal exposure	6,786.00 (45.91%)	4,723.00 (46.19%)
High metal exposure	9,137.00 (54.09%)	5,918.00 (53.81%)
Age, years	43.0 (25.0, 56.0)	45.0 (32.0, 56.0)
Sex, N (%)		
Female	7,554.00 (49.38%)	5,138.00 (49.53%)
Male	8,369.00 (50.62%)	5,503.00 (50.47%)
Race, N (%)		
White	6,567.00 (69.17%)	4,947.00 (70.91%)
Black	3,739.00 (11.12%)	2,227.00 (10.71%)
Mexican American	3,190.00 (7.97%)	1,938.00 (7.49%)
Other Hispanic	1,202.00 (5.58%)	789.00 (5.27%)
Other/multiracial	1,225.00 (6.16%)	740.00 (5.62%)
Family poverty income ratio (PIR)		
≤1.00	3,669.00 (14.61%)	2,172.00 (13.71%)
1.01-3.00	6,619.00 (36.20%)	4,390.00 (35.48%)
>3.00	5,635.00 (49.19%)	4,079.00 (50.81%)
Marital status, N (%)		
Married/Living with Partner	6,953.00 (54.46%)	6,309.00 (64.25%)
Widowed/Divorced/Separated	2,446.00 (15.56%)	2,118.00 (17.22%)
Never married	6,524.00 (29.98%)	2,214.00 (18.53%)
Urinary Creatinine, mg/dL	109.00 (61.00, 169.00)	109.00 (60.00, 171.00)
Diabetes status, N (%)		
Non-diabetes	9,849.00 (62.37%)	5,771.00 (59.74%)
Prediabetes	4,232.00 (27.66%)	3,351.00 (29.76%)
Diabetes	1,842.00 (9.97%)	1,519.00 (10.50%)
Body Mass Index (BMI), kg/m^2	26.57 (22.73, 30.89)	27.27 (23.90, 31.41)
Central obesity, N (%)	6,595.00 (45.70%)	5,430.00 (50.45%)
Barium (Ba), ng/mL	1.42 (0.70, 2.74)	1.38 (0.68, 2.60)
Cadmium (Cd), ng/mL	0.19 (0.09, 0.40)	0.23 (0.11, 0.45)
Cobalt (Co), ng/mL	0.37 (0.22, 0.57)	0.35 (0.21, 0.54)
Cesium (Cs), ng/mL	4.74 (2.77, 7.13)	4.73 (2.72, 7.16)
Molybdenum (Mo), ng/mL	42.90 (22.10, 75.10)	40.90 (21.15, 71.50)
Lead (Pb), ng/mL	0.50 (0.25, 0.88)	0.51 (0.26, 0.92)
Antimony (Sb), ng/mL	0.07 (0.04, 0.12)	0.07 (0.04, 0.12)
Thallium (Tl), ng/mL	0.17 (0.10, 0.26)	0.16 (0.09, 0.26)
Tungsten (Tu), ng/mL	0.07 (0.03, 0.14)	0.07 (0.03, 0.13)
Osteoporosis, N (%)	1,683.00 (12.68%)	1,415.00 (13.69%)

[1] N is unweighted, while percentage (%) and median (25th, 75th) are weighted.

[2] Median (25th, 75th) for continuous; N (%) for categorical

Supplementary Table S2. Basic characteristics of participants from two clusters (low-exposure and high-exposure group) identified by PAM clustering (N = 15923).

Characteristic [2]	Cluster 2<br>Low metal exposure,<br>N = 6786 (46%)[1]	Cluster 1<br>High metal exposure,<br>N = 9137 (54%)[1]	P
Age, years	46.0 (29.0, 58.0)	40.0 (23.0, 54.0)	<0.001
Sex, N (%)			<0.001
Female	3,628.00 (56.11%)	3,926.00 (43.66%)	
Male	3,158.00 (43.89%)	5,211.00 (56.34%)	
Race, N (%)			<0.001
White	3,041.00 (72.34%)	3,526.00 (66.49%)	
Black	1,336.00 (9.04%)	2,403.00 (12.89%)	
Mexican American	1,246.00 (7.29%)	1,944.00 (8.54%)	
Other Hispanic	554.00 (5.30%)	648.00 (5.82%)	
Other/multiracial	609.00 (6.04%)	616.00 (6.26%)	
Family poverty income ratio (PIR)	3.12 (1.58, 5.00)	2.77 (1.39, 4.69)	<0.001
Marital status, N (%)			<0.001
Married/Living with Partner	3,206.00 (56.67%)	3,747.00 (52.59%)	
Widowed/Divorced/Separated	1,185.00 (16.82%)	1,261.00 (14.50%)	
Never married	2,395.00 (26.51%)	4,129.00 (32.92%)	
Diabetes status, N (%)			0.1
Non-diabetes	3,983.00 (61.71%)	5,866.00 (62.93%)	
Prediabetes	1,883.00 (27.50%)	2,349.00 (27.79%)	
Diabetes	920.00 (10.78%)	922.00 (9.28%)	
General obesity (based on BMI, kg/m^2), N (%)			<0.001
Underweight (<18.5)	542.00 (5.45%)	941.00 (7.01%)	
Normal (18.5 to <25)	2,337.00 (34.64%)	3,126.00 (31.29%)	
Overweight (25 to <30)	2,096.00 (32.48%)	2,609.00 (31.49%)	
Obese (30 or greater)	1,811.00 (27.44%)	2,461.00 (30.20%)	
Central obesity, N (%)	3,015.00 (47.24%)	3,580.00 (44.40%)	0.013
Barium (Ba), ng/mL	0.90 (0.76, 1.03)	0.92 (0.79, 1.05)	<0.001
Cadmium (Cd), ng/mL	0.78 (0.43, 1.38)	2.17 (1.34, 3.74)	<0.001
Cobalt (Co), ng/mL	0.11 (0.05, 0.22)	0.29 (0.16, 0.56)	<0.001
Cesium (Cs), ng/mL	0.21 (0.13, 0.32)	0.51 (0.38, 0.72)	<0.001
Molybdenum (Mo), ng/mL	2.69 (1.74, 3.85)	6.60 (5.11, 8.70)	<0.001
Lead (Pb), ng/mL	22.20 (12.60, 35.40)	68.10 (47.20, 98.80)	<0.001
Antimony (Sb), ng/mL	0.25 (0.16, 0.42)	0.76 (0.50, 1.20)	<0.001
Thallium (Tl), ng/mL	0.04 (0.02, 0.06)	0.11 (0.07, 0.17)	<0.001
Tungsten (Tu), ng/mL	0.10 (0.06, 0.14)	0.24 (0.18, 0.32)	<0.001

[1] *N is unweighted, while percentage (%) and median (25th, 75th) are weighted.*
[2] *Median (25th, 75th) for continuous; N (%) for categorical*
Abbreviations: *BMI, body mass index.*

Supplementary Table S3. Comparison in characteristics of participants from two clusters (low-exposure and high-exposure group) identified by PAM clustering after PSM (N = 4850).

Characteristic [2]	Low urinary metal exposure N = 2425 (51%)[1]	High urinary metal exposure N=2425 (49%)[1]	P
Age, years	44.0 (26.0, 58.0)	45.0 (25.0, 58.0)	0.8
Sex, N (%)			0.9
Female	1,144.00 (50.05%)	1,140.00 (49.36%)	
Male	1,281.00 (49.95%)	1,285.00 (50.64%)	
Race, N (%)			0.5
White	975.00 (68.57%)	1,036.00 (70.77%)	
Black	557.00 (11.42%)	547.00 (10.57%)	
Mexican American	462.00 (8.25%)	524.00 (7.82%)	
Other Hispanic	206.00 (5.33%)	162.00 (5.22%)	
Other/multiracial	225.00 (6.43%)	156.00 (5.63%)	
Family poverty income ratio (PIR)	2.87 (1.46, 4.82)	2.91 (1.55, 4.85)	0.8
Urinary Creatinine, mg/dL	100.87 (81.00, 125.00)	102.00 (83.00, 124.00)	0.3
Diabetes status, N (%)			0.7
Non-diabetes	1,382.00 (59.83%)	1,501.00 (61.06%)	
Prediabetes	700.00 (28.56%)	620.00 (27.10%)	
Diabetes	343.00 (11.61%)	304.00 (11.84%)	
General obesity (based on BMI, kg/m^2), N (%)			0.5
Underweight (<18.5)	235.00 (7.09%)	242.00 (6.71%)	
Normal (18.5 to <25)	772.00 (31.17%)	861.00 (33.32%)	
Overweight (25 to <30)	721.00 (31.97%)	704.00 (32.32%)	
Obese (30 or greater)	697.00 (29.78%)	618.00 (27.65%)	
Central obesity, N (%)	1,081.00 (47.82%)	984.00 (45.88%)	0.4
Barium (Ba), ng/mL	1.00 (0.62, 1.76)	1.98 (1.28, 3.22)	<0.001
Cadmium (Cd), ng/mL	0.17 (0.09, 0.32)	0.24 (0.13, 0.45)	<0.001
Cobalt (Co), ng/mL	0.29 (0.21, 0.39)	0.45 (0.33, 0.62)	<0.001
Cesium (Cs), ng/mL	3.73 (2.86, 4.89)	5.51 (4.27, 7.09)	<0.001
Molybdenum (Mo), ng/mL	33.10 (22.90, 44.40)	57.30 (38.50, 80.14)	<0.001
Lead (Pb), ng/mL	0.34 (0.23, 0.51)	0.64 (0.43, 0.99)	<0.001
Antimony (Sb), ng/mL	0.05 (0.03, 0.07)	0.08 (0.05, 0.13)	<0.001
Thallium (Tl), ng/mL	0.13 (0.10, 0.18)	0.20 (0.15, 0.27)	<0.001
Tungsten (Tu), ng/mL	0.05 (0.03, 0.08)	0.09 (0.06, 0.16)	<0.001
Osteoporosis, N (%)	203.00 (9.59%)	331.00 (17.46%)	<0.001

[1] *N is unweighted, while percentage (%) and median (25th, 75th) are weighted.*
[2] *Median (25th, 75th) for continuous; N (%) for categorical*
[3] *Wilcoxon rank-sum test for complex survey samples; chi-squared test with Rao & Scott's second-order correction*

Supplementary Table S4. Associations of urinary metal exposure clusters (high vs low) identified by PAM clustering with osteopenia.

	N*	OR	95%CI	P value
**Model 1[1]**	15923	1.47	(1.28, 1.70)	<0.001
**Model 2[2]**	15923	1.30	(1.08, 1.56)	0.006
**Model 3[3]**	15923	1.25	(1.04, 1.50)	0.018
**Sensitivity analysis - PSM[4]**	4850	1.22	(1.01, 1.47)	0.043

Note： N is unweighted*

[1] *Model 1: Adjusted for urine creatinine*

[2] *Model 2: Adjusted for urine creatinine, age, sex, race/ethnicity, and poverty income ratio (PIR)*

[3] *Model 3: Additional adjustments included diabetes, general obesity, and central obesity*

[4] *PSM was utilised to ensure no statistical differences between the low-exposure and high-exposure clusters in the following variables: urine creatinine, age, sex, race/ethnicity, poverty income ratio (PIR), diabetes, general obesity, and central obesity. A univariate logistic regression model was conducted afterwards to calculate OR.*

Abbreviations*: OR odds ratio; 95% CI, 95% confidence interval*

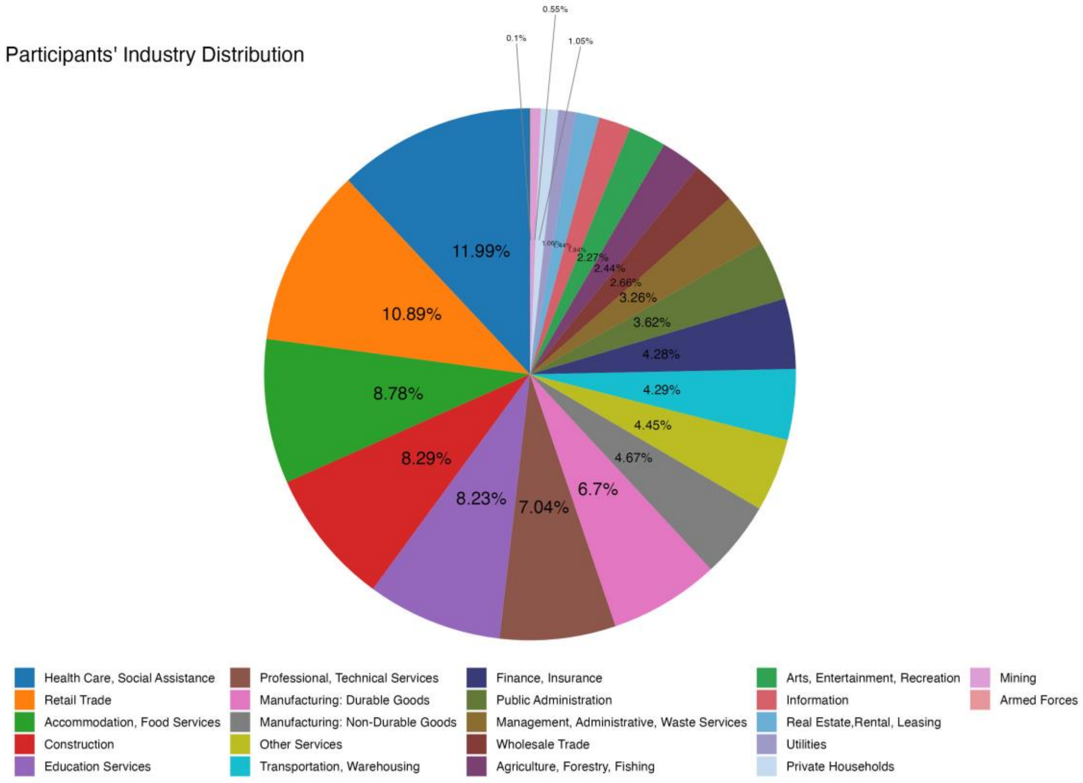

Supplementary Figure S3. The industry distribution of participants in this study.

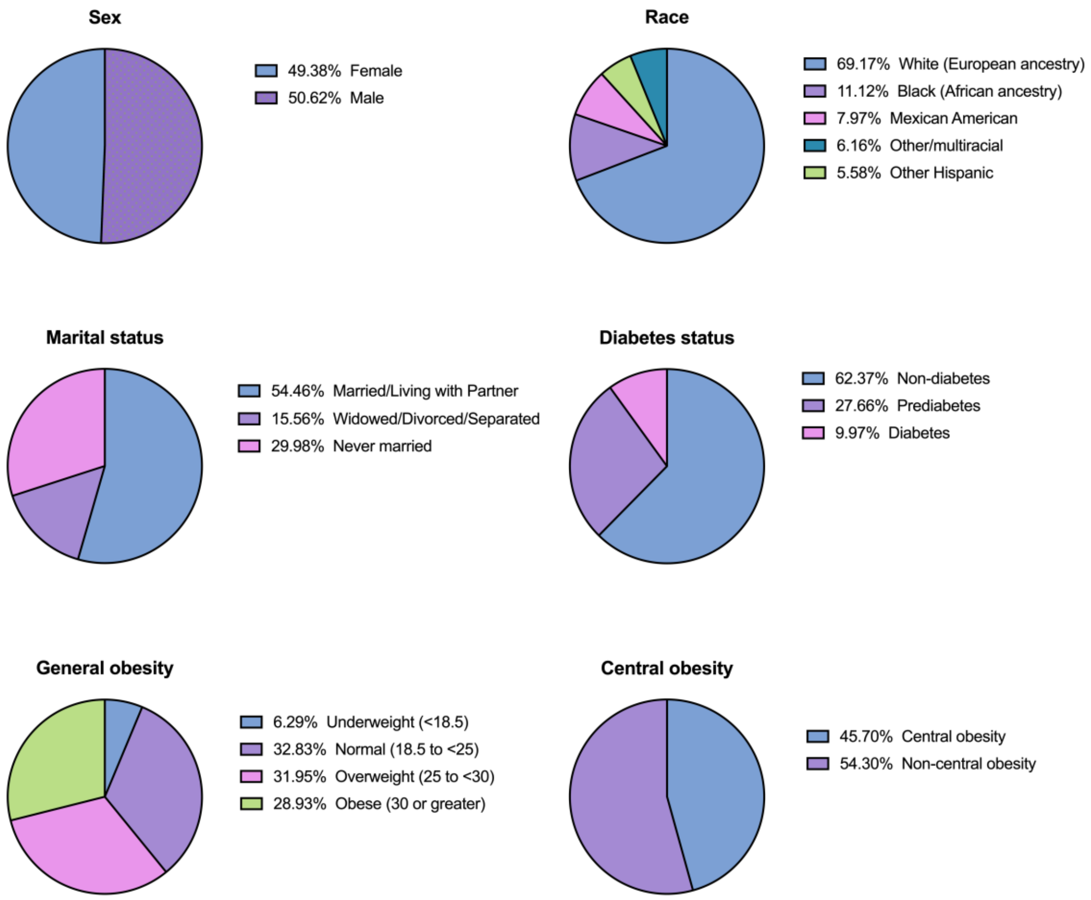

Supplementary Figure S4. Pie charts of the study participants' characteristics.

Supplementary Table S5. Results of Significance Tests for Temporal Differences in urinary Barium (Ba) Concentrations (1999–2020).

Shapiro-Wilk Test	TimeCycle	P value
	**1999-2000**	5.40852E-51
	**2001-2002**	4.89287E-70
	**2003-2004**	1.91504E-65
	**2005-2006**	2.49239E-58
	**2007-2008**	2.61517E-63
	**2009-2010**	6.95232E-64
	**2011-2012**	8.89842E-42
	**2013-2014**	1.45529E-48
	**2015-2016**	2.5669E-49
	**2017-2020**	1.62815E-43
**Levene's Test**	**P value**	
	7.23587E-10	
**Main Test Results**		
**Test Type**	**P value**	
**Kruskal-Wallis**	2.23809E-88	
**Posthoc Results**		
**Comparison**	**Z**	**P value**
**1999-2000 vs 2001-2002**	-1.105215416	0.269066258
**1999-2000 vs 2003-2004**	-0.33030326	0.74117083
**2001-2002 vs 2003-2004**	0.942143184	0.346119338
**1999-2000 vs 2005-2006**	-0.988186521	0.323061308
**2001-2002 vs 2005-2006**	0.112179421	0.910681146
**2003-2004 vs 2005-2006**	-0.800410308	0.423473111
**1999-2000 vs 2007-2008**	0.836712565	0.402754154
**2001-2002 vs 2007-2008**	2.304567792	0.021190792
**2003-2004 vs 2007-2008**	1.394482167	0.163172051
**2005-2006 vs 2007-2008**	2.128249008	0.033316443
**1999-2000 vs 2009-2010**	1.721664486	0.085130314
**2001-2002 vs 2009-2010**	3.413514775	0.000641307
**2003-2004 vs 2009-2010**	2.482766721	0.013036643
**2005-2006 vs 2009-2010**	3.19780875	0.001384761
**2007-2008 vs 2009-2010**	1.021322653	0.307101597
**1999-2000 vs 2011-2012**	3.946506545	7.92998E-05
**2001-2002 vs 2011-2012**	5.773656857	7.75693E-09
**2003-2004 vs 2011-2012**	4.955180098	7.22633E-07
**2005-2006 vs 2011-2012**	5.536756928	3.08124E-08
**2007-2008 vs 2011-2012**	3.604603722	0.00031263
**2009-2010 vs 2011-2012**	2.746966954	0.006014919
**1999-2000 vs 2013-2014**	10.24822426	1.20538E-24
**2001-2002 vs 2013-2014**	12.64778952	1.15067E-36
**2003-2004 vs 2013-2014**	11.92371245	8.90501E-33
**2005-2006 vs 2013-2014**	12.31996836	7.0722E-35
**2007-2008 vs 2013-2014**	10.59093867	3.28283E-26
**2009-2010 vs 2013-2014**	9.920678668	3.38447E-23
**2011-2012 vs 2013-2014**	6.902419153	5.11243E-12
**1999-2000 vs 2015-2016**	5.825843852	5.68247E-09
**2001-2002 vs 2015-2016**	8.027706674	9.93116E-16
**2003-2004 vs 2015-2016**	7.193060467	6.33549E-13
**2005-2006 vs 2015-2016**	7.727110545	1.10015E-14
**2007-2008 vs 2015-2016**	5.753723067	8.72991E-09
**2009-2010 vs 2015-2016**	4.919541614	8.67471E-07
**2011-2012 vs 2015-2016**	1.908174072	0.056368722
**2013-2014 vs 2015-2016**	-5.272441072	1.34621E-07
**1999-2000 vs 2017-2020**	11.43845776	2.68618E-30
**2001-2002 vs 2017-2020**	14.16552516	1.49743E-45
**2003-2004 vs 2017-2020**	13.41087305	5.22204E-41
**2005-2006 vs 2017-2020**	13.78245786	3.25015E-43
**2007-2008 vs 2017-2020**	11.97996579	4.5251E-33
**2009-2010 vs 2017-2020**	11.30882473	1.18668E-29
**2011-2012 vs 2017-2020**	8.036545016	9.2407E-16
**2013-2014 vs 2017-2020**	0.813149722	0.416132222
**2015-2016 vs 2017-2020**	6.366657734	1.93192E-10

Supplementary Table S6. Results of significance tests for temporal differences in urinary cadmium (Cd) concentrations (1999–2020).

Shapiro-Wilk Test	TimeCycle	P value
	**1999-2000**	5.81826E-50
	**2001-2002**	5.76853E-70
	**2003-2004**	6.63528E-62
	**2005-2006**	5.77917E-52
	**2007-2008**	4.80743E-51
	**2009-2010**	1.03459E-55
	**2011-2012**	3.8758E-52
	**2013-2014**	9.25399E-40
	**2015-2016**	5.30859E-48
	**2017-2020**	5.37936E-42
**Levene's Test**	**P value**	
	4.01393E-16	
**Main Test Results**		
**Test Type**	**P value**	
**Kruskal-Wallis**	4.8279E-171	
**Posthoc Results**		
**Comparison**	**Z**	**P value**
**1999-2000 vs 2001-2002**	4.652786498	3.27479E-06
**1999-2000 vs 2003-2004**	5.445449858	5.16746E-08
**2001-2002 vs 2003-2004**	0.962068975	0.336014957
**1999-2000 vs 2005-2006**	9.258965639	2.06422E-20
**2001-2002 vs 2005-2006**	5.662210602	1.49435E-08
**2003-2004 vs 2005-2006**	4.732131902	2.22174E-06
**1999-2000 vs 2007-2008**	6.550654512	5.72855E-11
**2001-2002 vs 2007-2008**	2.409788128	0.015961787
**2003-2004 vs 2007-2008**	1.480473085	0.138747038
**2005-2006 vs 2007-2008**	-3.146175453	0.001654207
**1999-2000 vs 2009-2010**	8.31058458	9.52318E-17
**2001-2002 vs 2009-2010**	4.471287809	7.775E-06
**2003-2004 vs 2009-2010**	3.521190584	0.000429614
**2005-2006 vs 2009-2010**	-1.270302464	0.203976911
**2007-2008 vs 2009-2010**	1.940538644	0.052314262
**1999-2000 vs 2011-2012**	16.40135817	1.8701E-60
**2001-2002 vs 2011-2012**	14.21447442	7.45087E-46
**2003-2004 vs 2011-2012**	13.38088069	7.82151E-41
**2005-2006 vs 2011-2012**	8.90767086	5.21158E-19
**2007-2008 vs 2011-2012**	11.73815812	8.12364E-32
**2009-2010 vs 2011-2012**	10.19496554	2.08819E-24
**1999-2000 vs 2013-2014**	2.848525849	0.004392229
**2001-2002 vs 2013-2014**	-1.134445959	0.25660753
**2003-2004 vs 2013-2014**	-1.876746284	0.060552876
**2005-2006 vs 2013-2014**	-5.536455398	3.08654E-08
**2007-2008 vs 2013-2014**	-2.995634017	0.002738749
**2009-2010 vs 2013-2014**	-4.591536256	4.39995E-06
**2011-2012 vs 2013-2014**	-12.58642321	2.50782E-36
**1999-2000 vs 2015-2016**	19.06226346	5.19768E-81
**2001-2002 vs 2015-2016**	17.34012964	2.34208E-67
**2003-2004 vs 2015-2016**	16.49034181	4.30507E-61
**2005-2006 vs 2015-2016**	11.8446957	2.29249E-32
**2007-2008 vs 2015-2016**	14.72543564	4.42608E-49
**2009-2010 vs 2015-2016**	13.2101607	7.66606E-40
**2011-2012 vs 2015-2016**	2.532091902	0.011338427
**2013-2014 vs 2015-2016**	15.0410078	3.95483E-51
**1999-2000 vs 2017-2020**	-1.752496381	0.079688492
**2001-2002 vs 2017-2020**	-6.553579072	5.61742E-11
**2003-2004 vs 2017-2020**	-7.328925291	2.32006E-13
**2005-2006 vs 2017-2020**	-11.02405592	2.92572E-28
**2007-2008 vs 2017-2020**	-8.371589086	5.68464E-17
**2009-2010 vs 2017-2020**	-10.11811933	4.59153E-24
**2011-2012 vs 2017-2020**	-17.92306296	7.79175E-72
**2013-2014 vs 2017-2020**	-4.47493201	7.64356E-06
**2015-2016 vs 2017-2020**	-20.5507677	7.57557E-94

Supplementary Table S7. Results of significance tests for temporal differences in urinary cobalt (Co) concentrations (1999–2020).

Shapiro-Wilk Test	TimeCycle	P value
	**1999-2000**	1.21659E-47
	**2001-2002**	2.32487E-53
	**2003-2004**	1.73615E-73
	**2005-2006**	4.09811E-65
	**2007-2008**	9.0746E-57
	**2009-2010**	3.02601E-68
	**2011-2012**	6.23749E-44
	**2013-2014**	6.23296E-44
	**2015-2016**	3.76999E-43
	**2017-2020**	1.94374E-54
**Levene's Test**	**P value**	
	0.602860263	
**Main Test Results**		
**Test Type**	**P value**	
**Kruskal-Wallis**	1.08105E-64	
**Posthoc Results**		
**Comparison**	**Z**	**P value**
**1999-2000 vs 2001-2002**	0.013588411	0.98915835
**1999-2000 vs 2003-2004**	5.418506918	6.00988E-08
**2001-2002 vs 2003-2004**	6.56912315	5.06124E-11
**1999-2000 vs 2005-2006**	0.512770312	0.608111999
**2001-2002 vs 2005-2006**	0.60092142	0.547892326
**2003-2004 vs 2005-2006**	-5.762198526	8.30253E-09
**1999-2000 vs 2007-2008**	3.674258658	0.000238541
**2001-2002 vs 2007-2008**	4.401885218	1.07314E-05
**2003-2004 vs 2007-2008**	-1.94751084	0.051473521
**2005-2006 vs 2007-2008**	3.690538521	0.00022378
**1999-2000 vs 2009-2010**	4.796063854	1.61814E-06
**2001-2002 vs 2009-2010**	5.791786317	6.96417E-09
**2003-2004 vs 2009-2010**	-0.70401655	0.481422467
**2005-2006 vs 2009-2010**	5.019394869	5.18345E-07
**2007-2008 vs 2009-2010**	1.246220168	0.212683577
**1999-2000 vs 2011-2012**	6.025782549	1.68293E-09
**2001-2002 vs 2011-2012**	7.016170703	2.28031E-12
**2003-2004 vs 2011-2012**	1.300551215	0.193412115
**2005-2006 vs 2011-2012**	6.320832692	2.60158E-10
**2007-2008 vs 2011-2012**	2.975290144	0.002927115
**2009-2010 vs 2011-2012**	1.903670626	0.056953094
**1999-2000 vs 2013-2014**	5.27329917	1.33993E-07
**2001-2002 vs 2013-2014**	5.962126339	2.48977E-09
**2003-2004 vs 2013-2014**	0.896448806	0.370013111
**2005-2006 vs 2013-2014**	5.37944074	7.47176E-08
**2007-2008 vs 2013-2014**	2.400333079	0.01638016
**2009-2010 vs 2013-2014**	1.435739898	0.151076368
**2011-2012 vs 2013-2014**	-0.234700878	0.814440906
**1999-2000 vs 2015-2016**	-2.716538305	0.006596856
**2001-2002 vs 2015-2016**	-3.201580796	0.001366757
**2003-2004 vs 2015-2016**	-9.037427965	1.60401E-19
**2005-2006 vs 2015-2016**	-3.659007404	0.000253194
**2007-2008 vs 2015-2016**	-7.054241718	1.73544E-12
**2009-2010 vs 2015-2016**	-8.330703158	8.03636E-17
**2011-2012 vs 2015-2016**	-9.259000481	2.06355E-20
**2013-2014 vs 2015-2016**	-8.120462757	4.64409E-16
**1999-2000 vs 2017-2020**	10.99425292	4.07272E-28
**2001-2002 vs 2017-2020**	12.56357511	3.34853E-36
**2003-2004 vs 2017-2020**	7.282324829	3.28116E-13
**2005-2006 vs 2017-2020**	11.81598018	3.22762E-32
**2007-2008 vs 2017-2020**	8.706022419	3.14725E-18
**2009-2010 vs 2017-2020**	7.797591483	6.30999E-15
**2011-2012 vs 2017-2020**	5.533167022	3.145E-08
**2013-2014 vs 2017-2020**	5.287749773	1.2383E-07
**2015-2016 vs 2017-2020**	14.27738817	3.02736E-46

Supplementary Table S8. Results of significance tests for temporal differences in urinary cesium (Cs) concentrations (1999–2020).

Shapiro-Wilk Test	TimeCycle	P value
	**1999-2000**	5.353E-40
	**2001-2002**	1.51349E-69
	**2003-2004**	1.22479E-71
	**2005-2006**	9.15727E-46
	**2007-2008**	2.38728E-50
	**2009-2010**	1.03954E-33
	**2011-2012**	1.04931E-34
	**2013-2014**	2.5567E-25
	**2015-2016**	1.63319E-49
	**2017-2020**	6.02175E-31
**Levene's Test**	**P value**	
	0.000153069	
**Main Test Results**		
**Test Type**	**P value**	
**Kruskal-Wallis**	9.76744E-49	
**Posthoc Results**		
**Comparison**	**Z**	**P value**
**1999-2000 vs 2001-2002**	-2.567912462	0.010231299
**1999-2000 vs 2003-2004**	-1.194626376	0.232233039
**2001-2002 vs 2003-2004**	1.669824539	0.094954084
**1999-2000 vs 2005-2006**	-0.936213814	0.349163112
**2001-2002 vs 2005-2006**	1.896614439	0.057878844
**2003-2004 vs 2005-2006**	0.279723974	0.779689284
**1999-2000 vs 2007-2008**	1.604286899	0.108650828
**2001-2002 vs 2007-2008**	4.946011979	7.57493E-07
**2003-2004 vs 2007-2008**	3.333285477	0.000858268
**2005-2006 vs 2007-2008**	2.963499486	0.003041626
**1999-2000 vs 2009-2010**	4.842370587	1.28299E-06
**2001-2002 vs 2009-2010**	8.951012807	3.52236E-19
**2003-2004 vs 2009-2010**	7.302372514	2.82737E-13
**2005-2006 vs 2009-2010**	6.79926489	1.05154E-11
**2007-2008 vs 2009-2010**	3.76408835	0.000167158
**1999-2000 vs 2011-2012**	5.926508794	3.09443E-09
**2001-2002 vs 2011-2012**	9.631050385	5.9122E-22
**2003-2004 vs 2011-2012**	8.180249552	2.83257E-16
**2005-2006 vs 2011-2012**	7.736650468	1.0207E-14
**2007-2008 vs 2011-2012**	5.046165676	4.50765E-07
**2009-2010 vs 2011-2012**	1.739859474	0.081883696
**1999-2000 vs 2013-2014**	5.559718535	2.7021E-08
**2001-2002 vs 2013-2014**	8.706783851	3.12618E-18
**2003-2004 vs 2013-2014**	7.420613708	1.16579E-13
**2005-2006 vs 2013-2014**	7.059728249	1.66829E-12
**2007-2008 vs 2013-2014**	4.664223514	3.09785E-06
**2009-2010 vs 2013-2014**	1.714703968	0.086399516
**2011-2012 vs 2013-2014**	0.156833574	0.875376012
**1999-2000 vs 2015-2016**	5.430025118	5.63461E-08
**2001-2002 vs 2015-2016**	9.142369081	6.10989E-20
**2003-2004 vs 2015-2016**	7.661672174	1.83528E-14
**2005-2006 vs 2015-2016**	7.2184315	5.25906E-13
**2007-2008 vs 2015-2016**	4.476126251	7.60095E-06
**2009-2010 vs 2015-2016**	1.094390197	0.273783882
**2011-2012 vs 2015-2016**	-0.613432239	0.539590574
**2013-2014 vs 2015-2016**	-0.712696311	0.4760337
**1999-2000 vs 2017-2020**	4.135333244	3.5444E-05
**2001-2002 vs 2017-2020**	7.241137333	4.44938E-13
**2003-2004 vs 2017-2020**	5.899592506	3.644E-09
**2005-2006 vs 2017-2020**	5.549308619	2.86802E-08
**2007-2008 vs 2017-2020**	3.057130904	0.002234667
**2009-2010 vs 2017-2020**	-0.038514062	0.96927782
**2011-2012 vs 2017-2020**	-1.511145119	0.130751481
**2013-2014 vs 2017-2020**	-1.532036681	0.125513383
**2015-2016 vs 2017-2020**	-0.958867831	0.337625332

Supplementary Table S9. Results of significance tests for temporal differences in urinary molybdenum (Mo) concentrations (1999–2020).

Shapiro-Wilk Test	TimeCycle	P value
	**1999-2000**	2.10198E-34
	**2001-2002**	7.21805E-46
	**2003-2004**	2.53909E-52
	**2005-2006**	3.35592E-45
	**2007-2008**	1.70254E-41
	**2009-2010**	5.02245E-48
	**2011-2012**	1.82022E-40
	**2013-2014**	3.62045E-34
	**2015-2016**	1.46489E-37
	**2017-2020**	2.67288E-39
**Levene's Test**	**P value**	
	2.59215E-17	
**Main Test Results**		
**Test Type**	**P value**	
**Kruskal-Wallis**	2.51657E-95	
**Posthoc Results**		
**Comparison**	**Z**	**P value**
**1999-2000 vs 2001-2002**	-0.061713178	0.950791246
**1999-2000 vs 2003-2004**	3.918794429	8.8993E-05
**2001-2002 vs 2003-2004**	4.837918583	1.31206E-06
**1999-2000 vs 2005-2006**	0.511573809	0.60894932
**2001-2002 vs 2005-2006**	0.688127922	0.491372228
**2003-2004 vs 2005-2006**	-3.997996647	6.38809E-05
**1999-2000 vs 2007-2008**	2.216521913	0.026655779
**2001-2002 vs 2007-2008**	2.737605791	0.006188821
**2003-2004 vs 2007-2008**	-1.938655863	0.052543256
**2005-2006 vs 2007-2008**	1.990629764	0.046521606
**1999-2000 vs 2009-2010**	4.554187824	5.25884E-06
**2001-2002 vs 2009-2010**	5.589387926	2.27871E-08
**2003-2004 vs 2009-2010**	0.80591789	0.420290195
**2005-2006 vs 2009-2010**	4.736885276	2.17028E-06
**2007-2008 vs 2009-2010**	2.697782781	0.006980297
**1999-2000 vs 2011-2012**	4.904311178	9.37558E-07
**2001-2002 vs 2011-2012**	5.787354798	7.15034E-09
**2003-2004 vs 2011-2012**	1.578173924	0.114525659
**2005-2006 vs 2011-2012**	5.045017415	4.5348E-07
**2007-2008 vs 2011-2012**	3.238005127	0.001203686
**2009-2010 vs 2011-2012**	0.86163551	0.388888124
**1999-2000 vs 2013-2014**	12.65819541	1.0079E-36
**2001-2002 vs 2013-2014**	14.40011274	5.16592E-47
**2003-2004 vs 2013-2014**	10.67149951	1.38375E-26
**2005-2006 vs 2013-2014**	13.58906016	4.65013E-42
**2007-2008 vs 2013-2014**	11.96935937	5.14241E-33
**2009-2010 vs 2013-2014**	9.975687334	1.94748E-23
**2011-2012 vs 2013-2014**	8.49740029	1.93884E-17
**1999-2000 vs 2015-2016**	6.258897234	3.87709E-10
**2001-2002 vs 2015-2016**	7.409257388	1.27009E-13
**2003-2004 vs 2015-2016**	3.114051857	0.001845371
**2005-2006 vs 2015-2016**	6.609114422	3.86626E-11
**2007-2008 vs 2015-2016**	4.764302471	1.89508E-06
**2009-2010 vs 2015-2016**	2.36914879	0.017829079
**2011-2012 vs 2015-2016**	1.343057779	0.179253265
**2013-2014 vs 2015-2016**	-7.398957269	1.37258E-13
**1999-2000 vs 2017-2020**	13.06727203	5.06495E-39
**2001-2002 vs 2017-2020**	15.00862122	6.4474E-51
**2003-2004 vs 2017-2020**	11.12047015	9.9745E-29
**2005-2006 vs 2017-2020**	14.13972458	2.16129E-45
**2007-2008 vs 2017-2020**	12.45216576	1.36101E-35
**2009-2010 vs 2017-2020**	10.38881291	2.78801E-25
**2011-2012 vs 2017-2020**	8.79241772	1.46375E-18
**2013-2014 vs 2017-2020**	-0.007005279	0.994410642
**2015-2016 vs 2017-2020**	7.662251322	1.82702E-14

Supplementary Table S10. Results of significance tests for temporal differences in urinary lead (Pb) concentrations (1999–2020).

Shapiro-Wilk Test	TimeCycle	P value
	**1999-2000**	5.29368E-45
	**2001-2002**	2.85924E-53
	**2003-2004**	5.81989E-54
	**2005-2006**	9.3274E-60
	**2007-2008**	5.56867E-59
	**2009-2010**	1.6054E-67
	**2011-2012**	1.17473E-58
	**2013-2014**	2.69851E-42
	**2015-2016**	7.95763E-54
	**2017-2020**	1.5056E-41
**Levene's Test**	**P value**	
	7.90737E-65	
**Main Test Results**		
**Test Type**	**P value**	
**Kruskal-Wallis**	0	
**Posthoc Results**		
**Comparison**	**Z**	**P value**
**1999-2000 vs 2001-2002**	5.596536928	2.18676E-08
**1999-2000 vs 2003-2004**	6.690365176	2.22614E-11
**2001-2002 vs 2003-2004**	1.327833898	0.184232986
**1999-2000 vs 2005-2006**	11.79699533	4.04498E-32
**2001-2002 vs 2005-2006**	7.604738048	2.85482E-14
**2003-2004 vs 2005-2006**	6.320988035	2.59896E-10
**1999-2000 vs 2007-2008**	14.75159105	3.00496E-49
**2001-2002 vs 2007-2008**	11.16160292	6.28481E-29
**2003-2004 vs 2007-2008**	9.8815245	5.00654E-23
**2005-2006 vs 2007-2008**	3.466937638	0.000526424
**1999-2000 vs 2009-2010**	18.4010355	1.28878E-75
**2001-2002 vs 2009-2010**	15.55655205	1.43631E-54
**2003-2004 vs 2009-2010**	14.24843738	4.58424E-46
**2005-2006 vs 2009-2010**	7.552325342	4.27555E-14
**2007-2008 vs 2009-2010**	4.002306648	6.27279E-05
**1999-2000 vs 2011-2012**	25.57657951	2.7803E-144
**2001-2002 vs 2011-2012**	23.92130973	1.8384E-126
**2003-2004 vs 2011-2012**	22.77193	8.7028E-115
**2005-2006 vs 2011-2012**	16.6780222	1.89386E-62
**2007-2008 vs 2011-2012**	13.51896589	1.20863E-41
**2009-2010 vs 2011-2012**	10.14739186	3.40337E-24
**1999-2000 vs 2013-2014**	22.90684613	3.9707E-116
**2001-2002 vs 2013-2014**	20.70770074	2.95215E-95
**2003-2004 vs 2013-2014**	19.68780082	2.74344E-86
**2005-2006 vs 2013-2014**	14.37332556	7.60881E-47
**2007-2008 vs 2013-2014**	11.56354604	6.30502E-31
**2009-2010 vs 2013-2014**	8.519455005	1.60306E-17
**2011-2012 vs 2013-2014**	-0.451811599	0.651404713
**1999-2000 vs 2015-2016**	32.20001071	1.7643E-227
**2001-2002 vs 2015-2016**	31.73389923	5.296E-221
**2003-2004 vs 2015-2016**	30.56317649	3.7788E-205
**2005-2006 vs 2015-2016**	24.14245206	8.9621E-129
**2007-2008 vs 2015-2016**	20.90965669	4.37368E-97
**2009-2010 vs 2015-2016**	17.6106201	2.04196E-69
**2011-2012 vs 2015-2016**	6.54201973	6.06935E-11
**2013-2014 vs 2015-2016**	6.363502005	1.97205E-10
**1999-2000 vs 2017-2020**	22.69459704	5.0657E-114
**2001-2002 vs 2017-2020**	20.48997742	2.64516E-93
**2003-2004 vs 2017-2020**	19.42650725	4.60662E-84
**2005-2006 vs 2017-2020**	13.88237833	8.10106E-44
**2007-2008 vs 2017-2020**	10.95723178	6.13472E-28
**2009-2010 vs 2017-2020**	7.782345532	7.11921E-15
**2011-2012 vs 2017-2020**	-1.473563944	0.140599035
**2013-2014 vs 2017-2020**	-0.920613684	0.357252155
**2015-2016 vs 2017-2020**	-7.616771548	2.60099E-14

Supplementary Table S11. Results of significance tests for temporal differences in urinary antimony (Sb) concentrations (1999–2020).

Shapiro-Wilk Test	TimeCycle	P value
	**1999-2000**	1.4249E-49
	**2001-2002**	3.63631E-66
	**2003-2004**	2.66071E-63
	**2005-2006**	1.07667E-67
	**2007-2008**	4.25391E-57
	**2009-2010**	2.10936E-65
	**2011-2012**	3.16549E-55
	**2013-2014**	2.06857E-52
	**2015-2016**	8.16004E-58
	**2017-2020**	1.38204E-54
**Levene's Test**	**P value**	
	5.54664E-23	
**Main Test Results**		
**Test Type**	**P value**	
**Kruskal-Wallis**	0	
**Posthoc Results**		
**Comparison**	**Z**	**P value**
**1999-2000 vs 2001-2002**	-0.556675859	0.577748914
**1999-2000 vs 2003-2004**	14.71736428	4.98723E-49
**2001-2002 vs 2003-2004**	18.56419697	6.26163E-77
**1999-2000 vs 2005-2006**	19.35518622	1.84327E-83
**2001-2002 vs 2005-2006**	23.94169847	1.1276E-126
**2003-2004 vs 2005-2006**	5.966926402	2.41764E-09
**1999-2000 vs 2007-2008**	26.3531938	4.7176E-153
**2001-2002 vs 2007-2008**	32.34055948	1.8833E-229
**2003-2004 vs 2007-2008**	14.40378028	4.8989E-47
**2005-2006 vs 2007-2008**	8.197907614	2.44607E-16
**1999-2000 vs 2009-2010**	30.71321111	3.792E-207
**2001-2002 vs 2009-2010**	37.86342134	0
**2003-2004 vs 2009-2010**	19.51374589	8.39028E-85
**2005-2006 vs 2009-2010**	13.00478132	1.14927E-38
**2007-2008 vs 2009-2010**	4.619746781	3.84209E-06
**1999-2000 vs 2011-2012**	27.88718373	3.8164E-171
**2001-2002 vs 2011-2012**	33.1260497	1.2533E-240
**2003-2004 vs 2011-2012**	16.97734156	1.20837E-64
**2005-2006 vs 2011-2012**	11.33435681	8.86812E-30
**2007-2008 vs 2011-2012**	3.906884585	9.34938E-05
**2009-2010 vs 2011-2012**	-0.191390731	0.848219481
**1999-2000 vs 2013-2014**	34.10277634	6.7093E-255
**2001-2002 vs 2013-2014**	39.16172978	0
**2003-2004 vs 2013-2014**	24.85085752	2.5313E-136
**2005-2006 vs 2013-2014**	19.71380424	1.6415E-86
**2007-2008 vs 2013-2014**	13.08691534	3.91174E-39
**2009-2010 vs 2013-2014**	9.569575706	1.07345E-21
**2011-2012 vs 2013-2014**	8.985248956	2.58149E-19
**1999-2000 vs 2015-2016**	30.41348608	3.6435E-203
**2001-2002 vs 2015-2016**	36.28058099	3.2753E-288
**2003-2004 vs 2015-2016**	19.80105335	2.91534E-87
**2005-2006 vs 2015-2016**	13.96820259	2.437E-44
**2007-2008 vs 2015-2016**	6.391596629	1.64162E-10
**2009-2010 vs 2015-2016**	2.257616619	0.023969572
**2011-2012 vs 2015-2016**	2.210800864	0.02704963
**2013-2014 vs 2015-2016**	-7.110017372	1.16028E-12
**1999-2000 vs 2017-2020**	34.12338753	3.3194E-255
**2001-2002 vs 2017-2020**	39.5796364	0
**2003-2004 vs 2017-2020**	24.65585753	3.1845E-134
**2005-2006 vs 2017-2020**	19.29073571	6.42525E-83
**2007-2008 vs 2017-2020**	12.39152523	2.90466E-35
**2009-2010 vs 2017-2020**	8.72296884	2.71001E-18
**2011-2012 vs 2017-2020**	8.15859004	3.38958E-16
**2013-2014 vs 2017-2020**	-1.049732117	0.293841293
**2015-2016 vs 2017-2020**	6.207264648	5.39148E-10

Supplementary Table S12. Results of significance tests for temporal differences in urinary thallium (Tl) concentrations (1999–2020).

Shapiro-Wilk Test	TimeCycle	P value
	**1999-2000**	4.27959E-25
	**2001-2002**	5.49337E-48
	**2003-2004**	6.63537E-37
	**2005-2006**	4.23629E-35
	**2007-2008**	9.44496E-51
	**2009-2010**	4.31594E-44
	**2011-2012**	7.55754E-33
	**2013-2014**	8.55102E-29
	**2015-2016**	1.93748E-56
	**2017-2020**	3.64507E-34
**Levene's Test**	**P value**	
	9.86862E-09	
**Main Test Results**		
**Test Type**	**P value**	
**Kruskal-Wallis**	6.42328E-58	
**Posthoc Results**		
**Comparison**	**Z**	**P value**
**1999-2000 vs 2001-2002**	2.677496622	0.00741746
**1999-2000 vs 2003-2004**	4.886320257	1.02738E-06
**2001-2002 vs 2003-2004**	2.683833002	0.007278345
**1999-2000 vs 2005-2006**	4.310605972	1.62808E-05
**2001-2002 vs 2005-2006**	2.03413861	0.041937622
**2003-2004 vs 2005-2006**	-0.564947087	0.572109755
**1999-2000 vs 2007-2008**	9.578479899	9.84837E-22
**2001-2002 vs 2007-2008**	8.371157573	5.7055E-17
**2003-2004 vs 2007-2008**	5.779232815	7.5042E-09
**2005-2006 vs 2007-2008**	6.154881548	7.51336E-10
**1999-2000 vs 2009-2010**	10.12582571	4.24382E-24
**2001-2002 vs 2009-2010**	9.044017166	1.51018E-19
**2003-2004 vs 2009-2010**	6.392413343	1.63288E-10
**2005-2006 vs 2009-2010**	6.753295282	1.44524E-11
**2007-2008 vs 2009-2010**	0.463706256	0.642858215
**1999-2000 vs 2011-2012**	5.615624821	1.95853E-08
**2001-2002 vs 2011-2012**	3.719744665	0.000199424
**2003-2004 vs 2011-2012**	1.384837865	0.16610205
**2005-2006 vs 2011-2012**	1.846556156	0.064811494
**2007-2008 vs 2011-2012**	-3.719834666	0.000199353
**2009-2010 vs 2011-2012**	-4.203810449	2.62459E-05
**1999-2000 vs 2013-2014**	11.07493095	1.66008E-28
**2001-2002 vs 2013-2014**	10.03822566	1.03519E-23
**2003-2004 vs 2013-2014**	7.970203739	1.58413E-15
**2005-2006 vs 2013-2014**	8.258446373	1.47581E-16
**2007-2008 vs 2013-2014**	3.290905502	0.000998655
**2009-2010 vs 2013-2014**	2.965426856	0.003022632
**2011-2012 vs 2013-2014**	6.179023528	6.44993E-10
**1999-2000 vs 2015-2016**	4.724499388	2.30683E-06
**2001-2002 vs 2015-2016**	2.652130666	0.007998558
**2003-2004 vs 2015-2016**	0.268967044	0.787955044
**2005-2006 vs 2015-2016**	0.767159811	0.442986504
**2007-2008 vs 2015-2016**	-4.906006826	9.29493E-07
**2009-2010 vs 2015-2016**	-5.422876683	5.86475E-08
**2011-2012 vs 2015-2016**	-1.02225328	0.30666104
**2013-2014 vs 2015-2016**	-7.184707131	6.73514E-13
**1999-2000 vs 2017-2020**	9.937663049	2.85445E-23
**2001-2002 vs 2017-2020**	8.750511261	2.12388E-18
**2003-2004 vs 2017-2020**	6.593656929	4.29123E-11
**2005-2006 vs 2017-2020**	6.915729578	4.65462E-12
**2007-2008 vs 2017-2020**	1.745721533	0.080859352
**2009-2010 vs 2017-2020**	1.385089644	0.166025058
**2011-2012 vs 2017-2020**	4.835587023	1.32753E-06
**2013-2014 vs 2017-2020**	-1.431305054	0.152342806
**2015-2016 vs 2017-2020**	5.861863792	4.577E-09

Supplementary Table S13. Results of significance tests for temporal differences in urinary tungsten (Tu) concentrations (1999–2020)

Shapiro-Wilk Test	TimeCycle	P value
	**1999-2000**	8.5995E-56
	**2001-2002**	2.99913E-68
	**2003-2004**	6.82676E-64
	**2005-2006**	1.20229E-67
	**2007-2008**	2.41234E-69
	**2009-2010**	2.42251E-63
	**2011-2012**	6.03314E-62
	**2013-2014**	2.43245E-48
	**2015-2016**	8.09612E-56
	**2017-2020**	8.10227E-54
**Levene's Test**	**P value**	
	8.01972E-05	
**Main Test Results**		
**Test Type**	**P value**	
**Kruskal-Wallis**	4.9437E-130	
**Posthoc Results**		
**Comparison**	**Z**	**P value**
**1999-2000 vs 2001-2002**	1.710651904	0.087145391
**1999-2000 vs 2003-2004**	4.20027186	2.66595E-05
**2001-2002 vs 2003-2004**	3.025389169	0.002483134
**1999-2000 vs 2005-2006**	-0.717002619	0.473372481
**2001-2002 vs 2005-2006**	-2.876433313	0.004021972
**2003-2004 vs 2005-2006**	-5.807978132	6.32318E-09
**1999-2000 vs 2007-2008**	-1.824339869	0.068100707
**2001-2002 vs 2007-2008**	-4.203399795	2.62936E-05
**2003-2004 vs 2007-2008**	-7.129621204	1.00646E-12
**2005-2006 vs 2007-2008**	-1.293492006	0.195840947
**1999-2000 vs 2009-2010**	3.846985113	0.00011958
**2001-2002 vs 2009-2010**	2.602443324	0.009256211
**2003-2004 vs 2009-2010**	-0.389200533	0.697127809
**2005-2006 vs 2009-2010**	5.369751743	7.88451E-08
**2007-2008 vs 2009-2010**	6.678507869	2.41387E-11
**1999-2000 vs 2011-2012**	1.89514118	0.0580737
**2001-2002 vs 2011-2012**	0.401619907	0.687963777
**2003-2004 vs 2011-2012**	-2.231436892	0.025652203
**2005-2006 vs 2011-2012**	2.914626414	0.003561146
**2007-2008 vs 2011-2012**	4.079457535	4.51409E-05
**2009-2010 vs 2011-2012**	-1.872886077	0.061084118
**1999-2000 vs 2013-2014**	14.38027685	6.88187E-47
**2001-2002 vs 2013-2014**	14.68972657	7.50159E-49
**2003-2004 vs 2013-2014**	12.35921795	4.34362E-35
**2005-2006 vs 2013-2014**	16.65795657	2.64922E-62
**2007-2008 vs 2013-2014**	17.67987773	5.9926E-70
**2009-2010 vs 2013-2014**	12.57777796	2.79789E-36
**2011-2012 vs 2013-2014**	13.13776383	2.00052E-39
**1999-2000 vs 2015-2016**	4.962541548	6.95767E-07
**2001-2002 vs 2015-2016**	3.975123185	7.03428E-05
**2003-2004 vs 2015-2016**	1.288938499	0.197419468
**2005-2006 vs 2015-2016**	6.447870607	1.13433E-10
**2007-2008 vs 2015-2016**	7.629757737	2.35195E-14
**2009-2010 vs 2015-2016**	1.623911961	0.1043946
**2011-2012 vs 2015-2016**	3.185787734	0.001443605
**2013-2014 vs 2015-2016**	-10.43808226	1.66133E-25
**1999-2000 vs 2017-2020**	13.8674223	9.97993E-44
**2001-2002 vs 2017-2020**	14.19119291	1.03877E-45
**2003-2004 vs 2017-2020**	11.76082249	6.21261E-32
**2005-2006 vs 2017-2020**	16.23845254	2.69641E-59
**2007-2008 vs 2017-2020**	17.30198811	4.54432E-67
**2009-2010 vs 2017-2020**	11.98930128	4.04301E-33
**2011-2012 vs 2017-2020**	12.5791306	2.7504E-36
**2013-2014 vs 2017-2020**	-0.939773231	0.347333893
**2015-2016 vs 2017-2020**	9.779098481	1.38438E-22

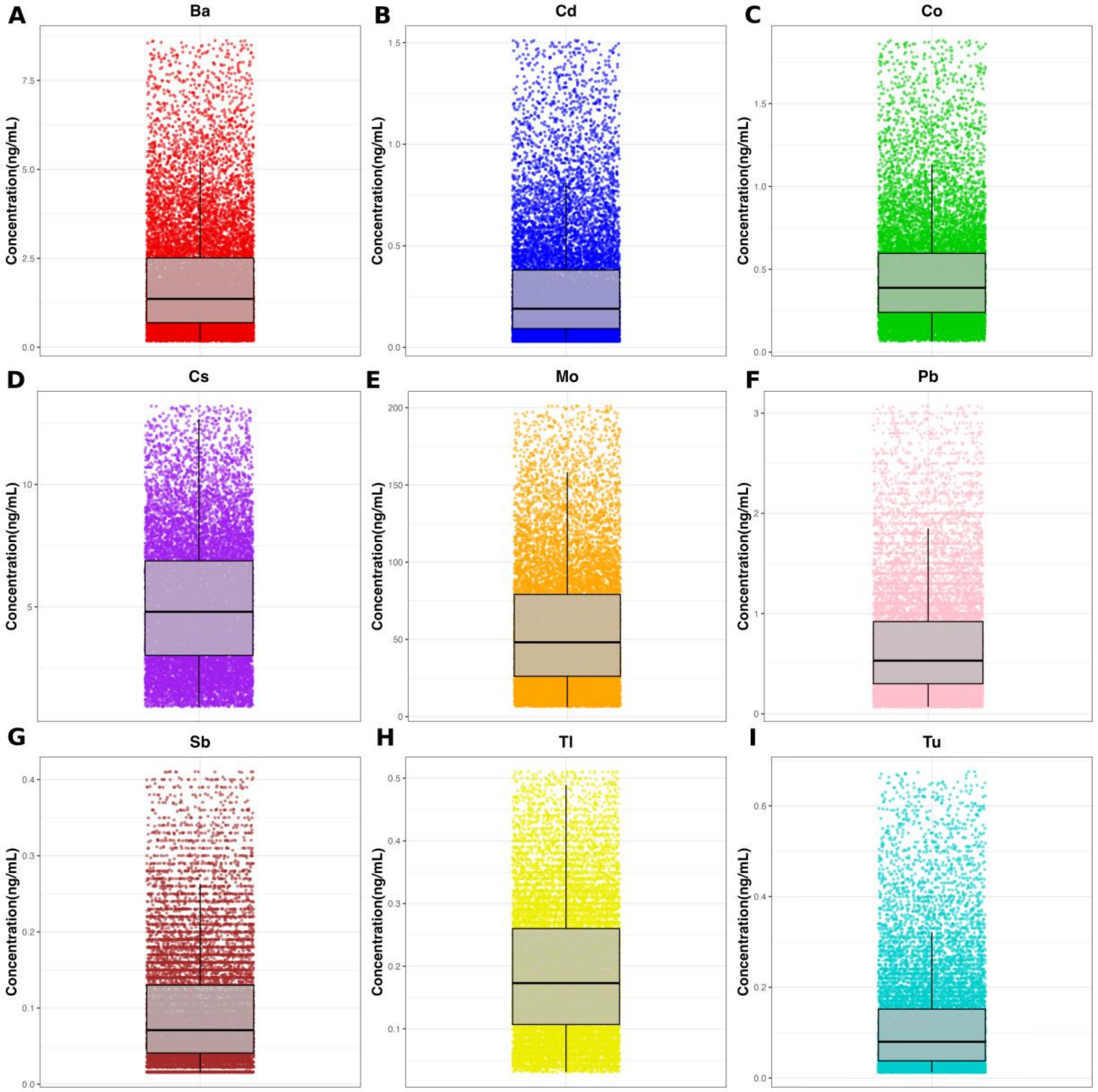

Supplementary Figure S5. Scatter and box plots illustrating the original urine metal concentration data (unweighted data) (N=15,923): (A) Barium (Ba), (B) Cadmium (Cd), (C) Cobalt (Co), (D) Cesium (Cs), (E) Molybdenum (Mo), (F) Lead (Pb), (G) Antimony (Sb), (H) Thallium (Tl), and (I) Tungsten (Tu). ***Note: data excluded upper 2.5% and lower 2.5% extreme values***
